# Crystal structures and hydrogen-bonding analysis of a series of solvated ammonium salts of molybdenum(II) chloride clusters

**DOI:** 10.1107/S205698901901380X

**Published:** 2019-10-22

**Authors:** Dean H. Johnston, Ikponmwosa Agho

**Affiliations:** a Department of Chemistry, Otterbein University, Westerville, OH 43081, USA

**Keywords:** metal–halide cluster, charge-assisted hydrogen bonds, primary ammonium salt, quaternary ammonium salt, Hirshfeld surface, crystal structure

## Abstract

Charge-assisted hydrogen bonding plays a significant role in the crystal structures of solvates of ionic com­pounds, especially when the cation or cations are primary ammonium salts. We report the crystal structures of four ammonium salts of molybdenum halide cluster solvates where we observe significant hydrogen bonding between the solvent molecules and cations. The crystal structures of bis­(anilinium) octa-μ_3_-chlorido-hexa­chlorido-*octa­hedro*-hexa­molybdate *N*,*N*-di­­methyl­formamide tetra­solvate, (C_6_H_8_N)_2_[Mo_6_Cl_8_Cl_6_]·4C_3_H_7_NO, (**I**), *p*-phenyl­enedi­ammonium octa-μ_3_-chlorido-hexa­chlorido-*octa­hedro*-hexa­mol­yb­date *N*,*N*-di­methyl­formamide hexa­solvate, (C_6_H_10_N_2_)[Mo_6_Cl_8_Cl_6_]·6C_3_H_7_NO, (**II**), *N*,*N*′-(1,4-phenyl­ene)bis­(propan-2-iminium) octa-μ_3_-chlorido-hexa­chlo­rido-*octa­hedro*-hexa­molybdate acetone tris­olvate, (C_12_H_18_N_2_)[Mo_6_Cl_8_Cl_6_]·3C_3_H_6_O, (**III**), and 1,1′-dimethyl-4,4′-bipyridinium octa-μ_3_-chlo­rido-hexa­chlorido-*octa­hedro*-hexa­molybdate *N*,*N*-di­methyl­formamide tetra­solvate, (C_12_H_14_N_2_)[Mo_6_Cl_8_Cl_6_]·4C_3_H_7_NO, (**IV**), are reported and described. In (**I**), the anilinium cations and *N*,*N*-di­methyl­formamide (DMF) solvent mol­ecules form a cyclic *R*_4_^2^(8) hydrogen-bonded motif centered on a crystallographic inversion center with an additional DMF mol­ecule forming a *D*(2) inter­action. The *p*-phenyl­enedi­ammonium cation in (**II**) forms three *D*(2) inter­actions between the three N—H bonds and three independent *N*,*N*-di­methyl­formamide mol­ecules. The dication in (**III**) is a protonated Schiff base solvated by acetone mol­ecules. Compound (**IV**) contains a methyl viologen dication with *N*,*N*-di­methyl­formamide mol­ecules forming close contacts with both aromatic and methyl H atoms.

## Abstract 2

The crystal structures of four different ammonium salts of the molybdenum halide cluster anion, [Mo_6_Cl_8_Cl_6_]^2−^, are reported. They display varying degrees of hydrogen bonding between ammonium ions and the respective solvent mol­ecules.

## Chemical context   

The unique photochemistry of the molybdenum and tungsten halide clusters [*M*_6_ *X*_8_ *Y*_6_]^2−^ (*M* = Mo, W; *X*, *Y* = Cl, Br, I) has been known for over 30 years (Maverick *et al.*, 1983 ▸) and researchers continue to explore the tunabilty of the redox potentials, crystal structures and photochemical properties of cluster-containing com­pounds *via* variation of the bridging and terminal ligands and the counter-ion (Mikhailov *et al.*, 2016 ▸; Saito *et al.*, 2017 ▸; Akagi *et al.*, 2018 ▸). Metal clusters, such as molybdenum halides, consist of an inner [Mo_6_ *X*_8_]^4+^ core surrounded by six axial ligands which are more labile than the core ligands, making the preparation of mixed-ligand cluster com­plexes relatively straightforward.

Charge-assisted hydrogen bonds (CAHBs) are particularly strong among hydrogen bonds (Gilli & Gilli, 2009 ▸) and can be a significant factor in the design and formation of supra­molecular com­plexes. CAHBs have been exploited in the formation of supra­molecular organic–inorganic uranyl materials (de Groot *et al.*, 2014 ▸), noncovalent macrocycles and catenanes (Pop *et al.*, 2016 ▸), mol­ecular switches (Gurbanov *et al.*, 2017 ▸), and CAHB networks (Ward, 2009 ▸). Protonated di­amines are a common motif found in hydrogen-bonded materials (Brozdowska & Chojnacki, 2017 ▸; Zick & Geiger, 2018 ▸). Examination of the nature and range of hydrogen bonding for solvates can provide information about the stability and physical properties of mol­ecular solids (Brychczynska *et al.*, 2012 ▸).

We have prepared a series of ammonium salts of the [Mo_6_Cl_8_Cl_6_]^2−^ com­plex anion, each containing cations ‘solvated’ by either di­methyl­formamide or acetone through strong CAHBs.

## Structural commentary   

**Figure 1:**
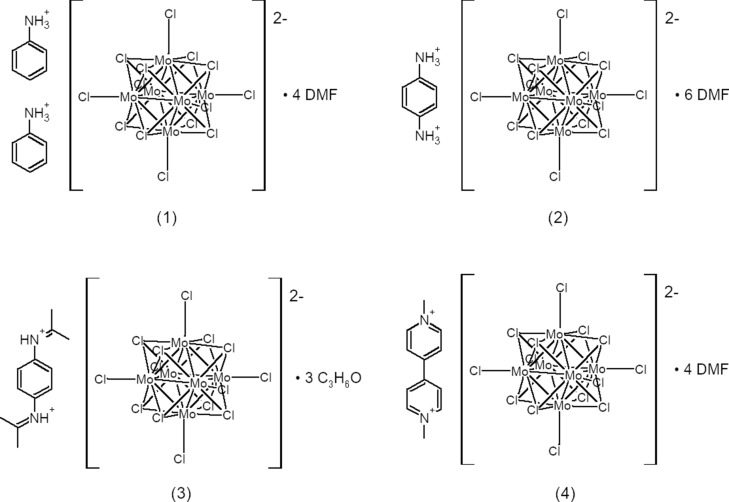
The structures of (I)–(IV).

**Figure 2:**
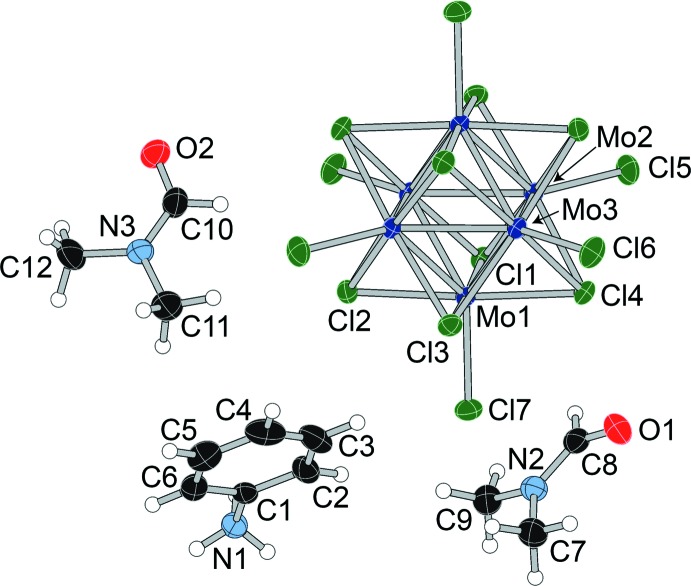
Displacement ellipsoid plot and atom-numbering scheme for (**I**), with ellipsoids drawn at the 50% probability level.

**Figure 3:**
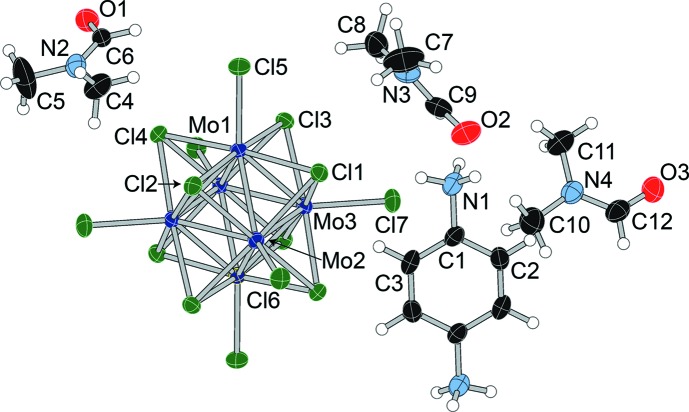
Displacement ellipsoid plot and atom-numbering scheme for (**II**), with ellipsoids drawn at the 50% probability level. The minor com­ponent of the disordered *p*-phenyl­enedi­ammonium cation is not shown for clarity.

The asymmetric unit of dianilinium salt (**I**) (Fig. 1 ▸) contains half a cluster unit, one anilinium cation, and two independent *N*,*N*-di­methyl­formamide (DMF) mol­ecules. The structure with the atom-numbering scheme is shown in Fig. 2 ▸. The [Mo_6_Cl_8_Cl_6_]^2−^ cluster unit resides on a crystallographic inversion center, as it does in all four structures. In com­pound (**II**), the asymmetric unit contains half a cluster unit, half a *p*-phenyl­enedi­ammonium cation, and three independent DMF mol­ecules. The *p*-phenyl­enedi­ammonium cation is disordered over two positions (rotation of 70.6° about the N—N axis), with a refined occupancy of 0.918 (4) for the primary orientation. The structure with the atom-numbering scheme is shown in Fig. 3 ▸.

**Figure 4:**
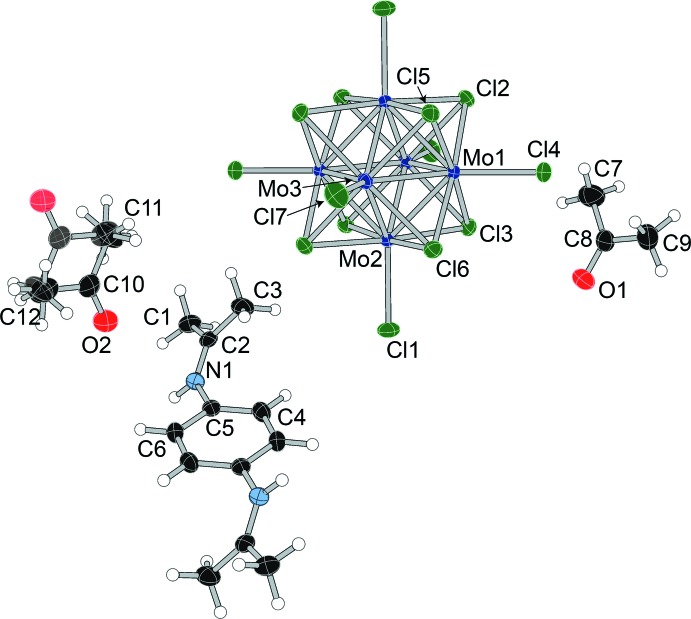
Displacement ellipsoid plot and atom-numbering scheme for (**III**), with ellipsoids drawn at the 50% probability level.

The asymmetric unit of Schiff base salt (**III**) contains half a cluster unit, half a Schiff base cation, and two independent acetone mol­ecules. The structure with the atom-numbering scheme is shown in Fig. 4 ▸. One acetone mol­ecule is disordered over an inversion center. The Schiff base cation, presumably formed from the reaction between a *p*-phenyl­enedi­ammonium cation and two acetone mol­ecules, shows strong similarities to the cation found in the bis­muthate structure reported by Shestimerova *et al.* (2018 ▸).

**Figure 5:**
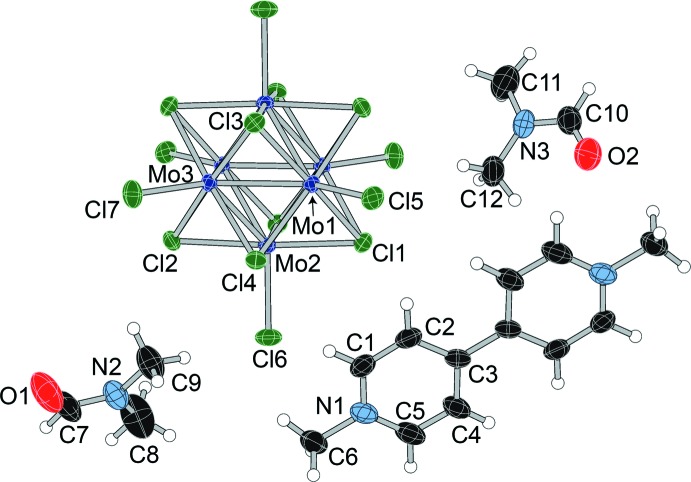
Displacement ellipsoid plot and atom-numbering scheme for (**IV**), with ellipsoids drawn at the 50% probability level.

For com­parison, a dicationic salt incapable of conventional hydrogen bonding (methyl viologen) was prepared and structurally characterized. The asymmetric unit of (**IV**), as in the other structures, contains half of the cluster unit, half of the methyl viologen dication, and two independent DMF mol­ecules. The structure with the atom-numbering scheme is shown in Fig. 5 ▸.

## Hydrogen-bonding analysis   

**Figure 6:**
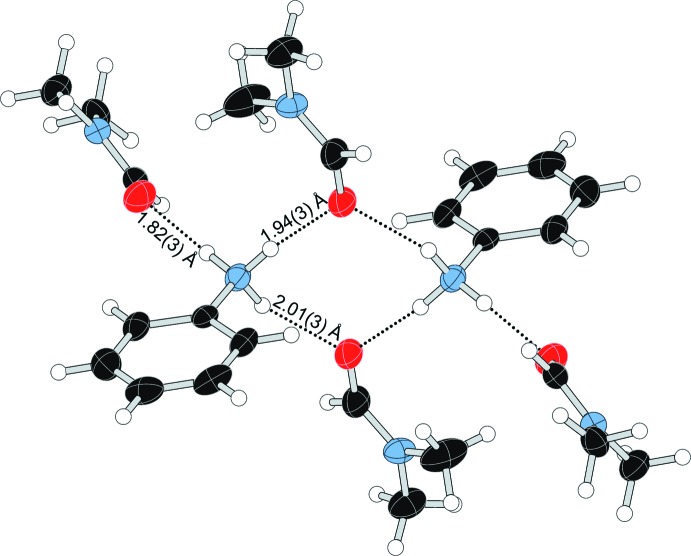
The cationic hydrogen-bonded dimer formed by anilinium cations and DMF mol­ecules in (**I**).

**Figure 7:**
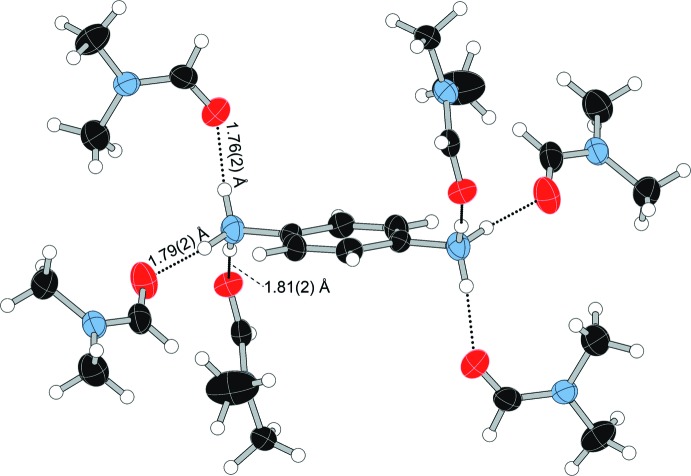
Hydrogen bonding in the DMF-solvated *p*-phenyl­enedi­ammonium dication in (**II**). The minor com­ponent of the disordered *p*-phenyl­enedi­ammonium cation is not shown for clarity.

**Figure 8:**
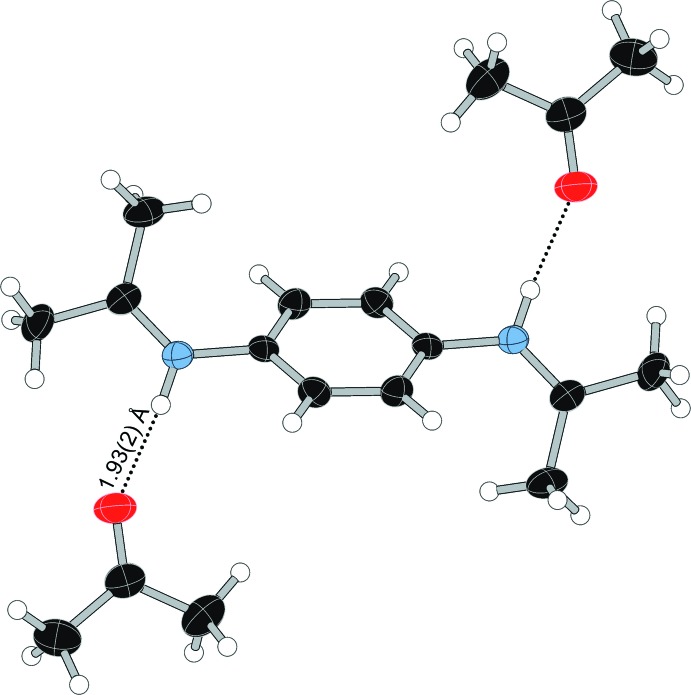
Hydrogen bonding in the acetone-solvated Schiff base dication in (**III**).

In com­pound (**I**), the anilinium cation and DMF mol­ecules form a cyclic 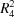
(8) hydrogen-bonded motif centered on a crystallographic inversion center, with an additional DMF forming a *D*(2) inter­action, as illustrated in Fig. 6 ▸. Although similar to some motifs discussed by Loehlin & Okasako (2007 ▸), the hydrogen-bonding network in (**I**) does not represent an example of saturated hydrogen bonding, as one DMF mol­ecule has an additional lone pair that is not involved in hydrogen bonding (Table 1 ▸). The DMF mol­ecules in com­pound (**II**) form three unique *D*(2) inter­actions with the three N—H bonds on each end of the *p*-phenyl­enedi­ammonium cations, as shown in Fig. 7 ▸ (Table 2 ▸). In com­pound (**III**), one acetone mol­ecule forms a hydrogen-bonding inter­action with the N—H group of the Schiff base, as shown in Fig. 8 ▸ (Table 3 ▸).

In spite of the lack of conventional hydrogen bonding in com­pound (**IV**), the methyl viologen cation forms several C—H⋯O contacts, with the O atoms of the two independent DMF mol­ecules forming close contacts with the H atoms of the aromatic ring (O⋯H = 2.23 Å) and the methyl group (O⋯H = 2.31 Å) (Table 4 ▸).

**Figure 9:**
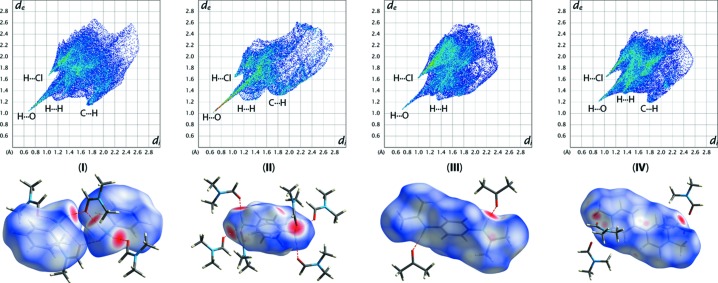
Fingerprint plots and Hirshfeld surfaces for (**I**)–(**IV**). For (**II**), only the major com­ponent of the disordered *p*-phenyl­enedi­ammonium cation was included in the generation of the fingerprint plot.

Analysis of the hydrogen bonding and close contacts *via* Hirshfeld surfaces and fingerprint plots was conducted using *CrystalExplorer* (Spackman & Jayatilaka, 2009 ▸) and the results are shown in Fig. 9 ▸. Compound (**II**) has the strongest hydrogen-bonding inter­actions, with similar, but slightly weaker, inter­actions for (**I**) and (**III**). All four com­pounds show very similar H(cation)⋯Cl(cluster anion) inter­actions. The C—H⋯O contacts in (**IV**), especially with the aromatic C—H group of the methyl viologen, can be clearly identified on the Hirshfeld surface.

## Database survey   

Inter­est in molybdenum(II) halide clusters and related com­pounds have led to numerous structural studies, with 45 entries in the Cambridge Structural Database (CSD, Version 5.40; Groom *et al.*, 2016 ▸) containing the [Mo_6_Cl_14_]^2−^ dianion and almost 200 structures containing the [Mo_6_ *X*_8_]^4+^ core. Similarly, one can find over 50 structures in the Inorganic Crystal Structure Database (ICSD, Version 4.2.0; Hellenbrandt, 2004 ▸) containing the same molybdenum halide core structure. The structures of the [Mo_6_Cl_14_]^2−^ cluster anions in this study are unremarkable and do not differ significantly from previous studies.

The anilinium cluster dihydrate structure published by Flemström (2003 ▸) has some similarities to (**I**). In that structure, the three N—H bonds of the anilinium cation serve as hydrogen-bond donors to one water mol­ecule (hydrate) and two terminal Cl atoms on two discrete cluster anions. The N—H⋯Cl inter­actions create 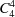
(15) chains. The water mol­ecules create 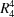
(14) rings involving two water mol­ecules and two cluster units, as well as 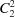
(8) and 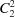
(7) chains.

While DMF-solvated ammonium salts appear to be relatively uncommon, a series of molybdenum halide cluster salts have been prepared with di­methyl­formamide-coordinated metal cations serving as the counter-cation (Khutornoi *et al.*, 2002 ▸; Kozhomuratova *et al.*, 2007 ▸; Liu *et al.*, 2006 ▸). The com­plexes prepared and characterized include the [Mo_6_Cl_8_Cl_6_]^2−^, [Mo_6_Br_8_Cl_6_]^2−^, and [Mo_6_Br_8_(NCS)_6_]^2−^ cluster anions as salts with [*M*(DMF)]^2+^ cations, where *M* = Ca^2+^, Mn^2+^, and Co^2+^. A similar set of rhenium chalcogenide cluster salts with DMF-solvated calcium and a series of lanthanides has been prepared by Perruchas *et al.* (2002 ▸) and Yarovoi *et al.* (2006 ▸).

A separate search of the CSD for structures with similar hydrogen-bonded networks containing anilinium and *p*-phenyl­enedi­ammonium cations yielded a large number of hits due to their propensity for forming significant hydrogen-bonding networks. In the structure of anilinium di­hydrogen phosphate (Kaman *et al.*, 2012 ▸), each of the three independent ammonium groups forms four different hydrogen bonds to the O atoms of nearby di­hydrogen phosphate moieties. A very similar set of hydrogen-bonding inter­actions and layered organic/inorganic structural arrangements are found in the structures of *p*-phenyl­enedi­ammonium bis­(di­hydrogen phosphate) (Mrad *et al.*, 2006*a* ▸) and *p*-phenyl­enedi­ammonium di­hydrogen diphosphate (Mrad *et al.*, 2006*b* ▸). While less closely related to the current report, the structure of *p*-phenyl­enedi­ammonium tetra­chlorido­zincate(II) (Bringley & Rajeswaran, 2006 ▸) also displays alternating organic and inorganic layers and strong hydrogen bonding between the tetra­chlorido­zinc(II) anions and the *p*-phenyl­enedi­ammonium cations.

A dimethyl sulfoxide (DMSO)-solvated *p*-phenyl­enedi­ammonium salt of an iodido­bis­muthate reported by Shestimerova *et al.* (2018 ▸) displays strong structural similarities to (**II**) in the way the DMSO solvates the *p*-phenyl­enedi­ammonium cation. Three unique DMSO mol­ecules also form *D*(2) inter­actions with each end of the *p*-phenyl­enedi­ammonium. One of the three DMSO mol­ecules simultaneously coordinates to one of the Bi atoms.

## Synthesis and crystallization   

All reagents were used as received from the manufacturer.

### Cluster synthesis, metathesis, and crystallization of (I), (II), and (IV)   

The hydro­nium salt of the [Mo_6_Cl_8_Cl_6_]^2−^ anion was prepared by the method of Hay *et al.* (2004 ▸) and then metathesized to the appropriate ammonium salt by combining an ethano­lic solution of (H_3_O)_2_[Mo_6_Cl_8_Cl_6_]·6H_2_O with a slight stoichiometric excess (∼2.5 times) of the respective ammonium chloride salt (anilinium chloride, *p*-phenyl­enedi­amine hydro­chloride, and methyl viologen dichloride). The bright-yellow precipitate that formed was isolated by filtration and the product was recrystallized by vapor diffusion of diethyl ether into a di­methyl­formamide solution of the cluster salt.

### Synthesis and crystallization of Schiff base salt (III)   

The cluster in com­pound (**III**) was prepared and metathesized to the di­ammonium salt *via* the same procedure as above using the *p*-phenyl­enedi­ammonium chloride to isolate a yellow precipitate. The salt was then redissolved in acetone and allowed to evaporate. The acetone inadvertently formed a Schiff base dication in a reaction with the *p*-phenyl­enedi­ammonium cation (Kolb & Bahadir, 1994 ▸).

## Refinement   

Crystal data, data collection, and structure refinement details are summarized in Table 5 ▸. All H atoms were located in a difference map. All carbon-bonded H atoms were placed in idealized positions using a riding model, with aromatic and amide C—H = 0.95 Å and methyl C—H = 0.98 Å, and with *U*_iso_(H) = 1.2*U*_eq_(C) (aromatic and amide) or *U*_iso_(H) = 1.5*U*_eq_(C) (meth­yl). The positions of all H atoms bonded to N atoms were refined with N—H distances restrained to 0.91 (2) (NH_3_) or 0.88 (2) Å (Schiff base), and with *U*_iso_(H) = 1.5*U*_eq_(N).

All four structures were refined in the space group *P* 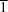 and the [Mo_6_Cl_14_]^2−^ dianion sits on an inversion center in every case. The dications in (**II**), (**III**), and (**IV**) are also each located on an inversion center. The *p*-phenyl­enedi­ammonium cation in (**II**) is disordered over two orientations with an occupancy of 0.918 (4) for the major com­ponent. One of the two acetone mol­ecules in (**III**) is disordered over an inversion center.

## Supplementary Material

Crystal structure: contains datablock(s) global, 1, 2, 3, 4. DOI: 10.1107/S205698901901380X/eb2023sup1.cif

Structure factors: contains datablock(s) 1. DOI: 10.1107/S205698901901380X/eb20231sup2.hkl

Supporting information file. DOI: 10.1107/S205698901901380X/eb20231sup6.mol

Structure factors: contains datablock(s) 2. DOI: 10.1107/S205698901901380X/eb20232sup3.hkl

Supporting information file. DOI: 10.1107/S205698901901380X/eb20232sup7.mol

Structure factors: contains datablock(s) 3. DOI: 10.1107/S205698901901380X/eb20233sup4.hkl

Supporting information file. DOI: 10.1107/S205698901901380X/eb20233sup8.mol

Structure factors: contains datablock(s) 4. DOI: 10.1107/S205698901901380X/eb20234sup5.hkl

Supporting information file. DOI: 10.1107/S205698901901380X/eb20234sup9.mol

CCDC references: 1907244, 1907230, 1907249, 1907231, 1907230, 1907231, 1907244, 1907249

Additional supporting information:  crystallographic information; 3D view; checkCIF report

## Figures and Tables

**Table 1:** Hydrogen-bond geometry (Å, °) for (**I**)

*D*—H⋯*A*	*D*—H	H⋯*A*	*D*⋯*A*	*D*—H⋯*A*
N1—H1*A*⋯O2^i^	0.89 (3)	2.01 (3)	2.827 (3)	152 (2)
N1—H1*B*⋯O2^ii^	0.91 (3)	1.94 (3)	2.833 (3)	168 (3)
N1—H1*C*⋯O1^iii^	0.91 (3)	1.82 (3)	2.715 (3)	166 (3)

Symmetry codes: (i) 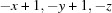; (ii) 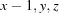; (iii) 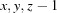.

**Table 2:** Hydrogen-bond geometry (Å, °) for (**II**)

*D*—H⋯*A*	*D*—H	H⋯*A*	*D*⋯*A*	*D*—H⋯*A*
N1—H1*A*⋯O2^i^	0.92 (2)	1.76 (2)	2.672 (4)	171 (4)
N1—H1*B*⋯O3^ii^	0.93 (2)	1.79 (2)	2.710 (4)	173 (4)
N1—H1*C*⋯O1^iii^	0.92 (2)	1.81 (2)	2.727 (4)	175 (4)

Symmetry codes: (i) 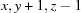; (ii) 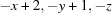; (iii) 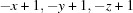.

**Table 3:** Hydrogen-bond geometry (Å, °) for (**III**)

*D*—H⋯*A*	*D*—H	H⋯*A*	*D*⋯*A*	*D*—H⋯*A*
N1—H1⋯O1^i^	0.87 (2)	1.93 (2)	2.791 (4)	172 (3)

Symmetry code: (i) 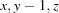.

**Table 4:** Hydrogen-bond geometry (Å, °) for (**IV**)

*D*—H⋯*A*	*D*—H	H⋯*A*	*D*⋯*A*	*D*—H⋯*A*
C5—H5⋯O1^i^	0.95	2.23	3.063 (4)	145
C6—H6*C*⋯O2^ii^	0.98	2.31	3.088 (4)	136

Symmetry codes: (i) 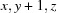; (ii) 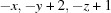.

**Table 5:** Experimental details

	(**I**)	(**II**)	(**III**)	(**IV**)
Crystal data				
Chemical formula	(C_6_H_8_N)_2_[Mo_6_Cl_8_Cl_6_]·4C_3_H_7_NO	(C_6_H_10_N_2_)[Mo_6_Cl_8_Cl_6_]·6C_3_H_7_NO	(C_12_H_18_N_2_)[Mo_6_Cl_8_Cl_6_]·3C_3_H_6_O	(C_12_H_14_N_2_)[Mo_6_Cl_8_Cl_6_]·4C_3_H_7_NO
*M*_r_	1552.59	1620.67	1436.46	1550.57
Crystal system, space group	Triclinic, *P* 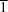	Triclinic, *P* 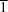	Triclinic, *P* 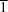	Triclinic, *P* 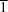
Temperature (K)	200	200	200	200
*a*, *b*, *c* (Å)	9.9813 (11), 10.6074 (13), 12.1686 (15)	10.1752 (16), 10.3227 (16), 13.736 (2)	9.451 (2), 11.236 (3), 11.712 (3)	9.8252 (11), 10.0933 (11), 12.6319 (15)
α, β, γ (°)	104.606 (3), 90.709 (3), 103.146 (3)	95.204 (4), 111.483 (4), 101.973 (4)	64.933 (6), 71.174 (6), 75.440 (6)	107.395 (3), 91.881 (3), 93.309 (3)
*V* (Å^3^)	1210.7 (3)	1291.1 (3)	1056.7 (5)	1191.8 (2)
*Z*	1	1	1	1
Radiation type	Mo *K*α	Mo *K*α	Mo *K*α	Mo *K*α
μ (mm^−1^)	2.32	2.18	2.64	2.35
Crystal size (mm)	0.48 × 0.46 × 0.12	0.50 × 0.13 × 0.13	0.55 × 0.33 × 0.20	0.32 × 0.30 × 0.28

Data collection				
Diffractometer	Bruker SMART X2S benchtop	Bruker SMART X2S benchtop	Bruker SMART X2S benchtop	Bruker SMART X2S benchtop
Absorption correction	Multi-scan (*SADABS*; Bruker, 2012 ▸)	Multi-scan (*SADABS*; Bruker, 2012 ▸)	Multi-scan (*SADABS*; Bruker, 2012 ▸)	Multi-scan (*SADABS*; Bruker, 2012 ▸)
*T*_min_, *T*_max_	0.498, 0.745	0.552, 0.745	0.490, 0.745	0.815, 1.000
No. of measured, independent and observed [*I* > 2σ(*I*)] reflections	11709, 4245, 3834	12459, 4504, 3666	10036, 3692, 3220	11498, 4187, 3743
*R*_int_	0.026	0.035	0.030	0.024
(sin θ/λ)_max_ (Å^−1^)	0.597	0.595	0.598	0.597

Refinement				
*R*[*F* ^2^ > 2σ(*F* ^2^)], *wR*(*F* ^2^), *S*	0.019, 0.046, 1.07	0.026, 0.062, 1.03	0.025, 0.068, 1.05	0.020, 0.049, 1.02
No. of reflections	4245	4504	3692	4187
No. of parameters	258	285	235	250
No. of restraints	0	144	13	0
H-atom treatment	H atoms treated by a mixture of independent and constrained refinement	H atoms treated by a mixture of independent and constrained refinement	H atoms treated by a mixture of independent and constrained refinement	H-atom parameters constrained
Δρ_max_, Δρ_min_ (e Å^−3^)	0.36, −0.54	0.66, −0.56	0.96, −0.82	0.42, −0.44

Computer programs: *APEX2* (Bruker, 2012 ▸), *SAINT* (Bruker, 2012 ▸), *SHELXS* (Sheldrick, 2008 ▸), *SHELXL2018* (Sheldrick, 2015 ▸), *CrystalMaker* (Palmer, 2019 ▸), *CrystalExplorer* (Spackman & Jayatilaka, 2009 ▸), *OLEX2* (Dolomanov *et al.*, 2009 ▸) and *publCIF* (Westrip, 2010 ▸).

## supplementary crystallographic information

### Bis(anilinium) octa-µ3-chlorido-hexachlorido-octahedro-hexamolybdate N,N-dimethylformamide tetrasolvate (1) Crystal data

**Table:**

(C_6_H_8_N)_2_[Mo_6_Cl_8_Cl_6_]·4C_3_H_7_NO	*Z* = 1
*M**_r_* = 1552.59	*F*(000) = 752
Triclinic, *P*1	*D*_x_ = 2.129 Mg m^−^^3^
*a* = 9.9813 (11) Å	Mo *K*α radiation, λ = 0.71073 Å
*b* = 10.6074 (13) Å	Cell parameters from 7006 reflections
*c* = 12.1686 (15) Å	θ = 2.3–25.1°
α = 104.606 (3)°	µ = 2.32 mm^−^^1^
β = 90.709 (3)°	*T* = 200 K
γ = 103.146 (3)°	Plate, clear orangish yellow
*V* = 1210.7 (3) Å^3^	0.48 × 0.46 × 0.12 mm

### Bis(anilinium) octa-µ3-chlorido-hexachlorido-octahedro-hexamolybdate N,N-dimethylformamide tetrasolvate (1) Data collection

**Table:**

Bruker SMART X2S benchtop diffractometer	4245 independent reflections
Radiation source: sealed microfocus source, XOS X-beam microfocus source	3834 reflections with *I* > 2σ(*I*)
Graphite monochromator	*R*_int_ = 0.026
Detector resolution: 8.3330 pixels mm^-1^	θ_max_ = 25.1°, θ_min_ = 2.3°
φ and ω scans	*h* = −11→11
Absorption correction: multi-scan (SADABS; Bruker, 2012)	*k* = −12→12
*T*_min_ = 0.498, *T*_max_ = 0.745	*l* = −14→14
11709 measured reflections	

### Bis(anilinium) octa-µ3-chlorido-hexachlorido-octahedro-hexamolybdate N,N-dimethylformamide tetrasolvate (1) Refinement

**Table:**

Refinement on *F*^2^	Hydrogen site location: mixed
Least-squares matrix: full	H atoms treated by a mixture of independent and constrained refinement
*R*[*F*^2^ > 2σ(*F*^2^)] = 0.019	*w* = 1/[σ^2^(*F*_o_^2^) + (0.0123*P*)^2^ + 0.5808*P*] where *P* = (*F*_o_^2^ + 2*F*_c_^2^)/3
*wR*(*F*^2^) = 0.046	(Δ/σ)_max_ = 0.001
*S* = 1.07	Δρ_max_ = 0.36 e Å^−^^3^
4245 reflections	Δρ_min_ = −0.54 e Å^−^^3^
258 parameters	Extinction correction: SHELXL2018 (Sheldrick, 2015), Fc^*^=kFc[1+0.001xFc^2^λ^3^/sin(2θ)]^-1/4^
0 restraints	Extinction coefficient: 0.0096 (3)
Primary atom site location: structure-invariant direct methods	

### Bis(anilinium) octa-µ3-chlorido-hexachlorido-octahedro-hexamolybdate N,N-dimethylformamide tetrasolvate (1) Special details

**Table:**

Geometry. All esds (except the esd in the dihedral angle between two l.s. planes) are estimated using the full covariance matrix. The cell esds are taken into account individually in the estimation of esds in distances, angles and torsion angles; correlations between esds in cell parameters are only used when they are defined by crystal symmetry. An approximate (isotropic) treatment of cell esds is used for estimating esds involving l.s. planes.

### Bis(anilinium) octa-µ3-chlorido-hexachlorido-octahedro-hexamolybdate N,N-dimethylformamide tetrasolvate (1) Fractional atomic coordinates and isotropic or equivalent isotropic displacement parameters (Å^2^)

**Table:**

	*x*	*y*	*z*	*U*_iso_*/*U*_eq_	
Mo1	0.33000 (2)	0.50142 (2)	0.43962 (2)	0.01755 (7)	
Mo2	0.42505 (2)	0.33816 (2)	0.52988 (2)	0.01753 (7)	
Mo3	0.45872 (2)	0.58737 (2)	0.64151 (2)	0.01766 (7)	
Cl1	0.30660 (6)	0.26453 (5)	0.33717 (5)	0.02208 (12)	
Cl2	0.44848 (6)	0.57046 (5)	0.27978 (5)	0.02298 (13)	
Cl3	0.37196 (6)	0.73926 (5)	0.55008 (5)	0.02352 (13)	
Cl4	0.22876 (5)	0.43149 (5)	0.60532 (5)	0.02318 (13)	
Cl5	0.32774 (6)	0.12583 (6)	0.57003 (5)	0.03234 (15)	
Cl6	0.39836 (6)	0.69501 (6)	0.82888 (5)	0.03331 (15)	
Cl7	0.10839 (6)	0.50315 (6)	0.35713 (5)	0.03118 (14)	
N1	0.1138 (2)	0.6861 (2)	−0.02394 (19)	0.0291 (5)	
H1A	0.144 (3)	0.612 (3)	−0.048 (2)	0.044*	
H1B	0.026 (3)	0.651 (3)	−0.010 (2)	0.044*	
H1C	0.111 (3)	0.732 (3)	−0.078 (2)	0.044*	
C1	0.1999 (2)	0.7715 (2)	0.0778 (2)	0.0262 (5)	
C2	0.1890 (3)	0.7327 (3)	0.1777 (2)	0.0352 (6)	
H2	0.125907	0.652029	0.180955	0.042*	
C3	0.2713 (3)	0.8128 (3)	0.2728 (2)	0.0439 (7)	
H3	0.264848	0.787222	0.342295	0.053*	
C4	0.3621 (3)	0.9288 (3)	0.2678 (3)	0.0454 (7)	
H4	0.418475	0.983410	0.333788	0.055*	
C5	0.2894 (3)	0.8879 (2)	0.0709 (2)	0.0326 (6)	
H5	0.294613	0.913873	0.001580	0.039*	
C6	0.3718 (3)	0.9666 (3)	0.1671 (2)	0.0411 (7)	
H6	0.435330	1.046973	0.163742	0.049*	
O1	0.0921 (2)	0.85206 (19)	0.84303 (16)	0.0431 (5)	
N2	0.0472 (2)	0.88716 (19)	0.67193 (17)	0.0287 (5)	
C7	0.0074 (3)	1.0112 (3)	0.7195 (2)	0.0408 (7)	
H7A	0.004355	1.025920	0.802189	0.061*	
H7B	−0.083924	1.006277	0.685843	0.061*	
H7C	0.074846	1.085755	0.702938	0.061*	
C8	0.0860 (2)	0.8186 (3)	0.7380 (2)	0.0325 (6)	
H8	0.111034	0.737608	0.702115	0.039*	
C9	0.0410 (3)	0.8403 (3)	0.5486 (2)	0.0364 (6)	
H9A	0.057014	0.749801	0.526886	0.055*	
H9B	0.111888	0.901063	0.519142	0.055*	
H9C	−0.050269	0.838466	0.516599	0.055*	
O2	0.85598 (19)	0.57853 (18)	0.05179 (17)	0.0418 (5)	
N3	0.7318 (2)	0.7329 (2)	0.05377 (17)	0.0320 (5)	
C10	0.7698 (3)	0.6417 (3)	0.0921 (2)	0.0397 (7)	
H10	0.727510	0.621423	0.157083	0.048*	
C11	0.6338 (3)	0.8071 (3)	0.1095 (2)	0.0460 (7)	
H11A	0.607218	0.779492	0.178708	0.069*	
H11B	0.551612	0.788223	0.057583	0.069*	
H11C	0.676785	0.903485	0.129568	0.069*	
C12	0.7879 (4)	0.7684 (4)	−0.0466 (3)	0.0621 (10)	
H12A	0.866691	0.846144	−0.023497	0.093*	
H12B	0.716767	0.790546	−0.089431	0.093*	
H12C	0.818232	0.692417	−0.094617	0.093*	

### Bis(anilinium) octa-µ3-chlorido-hexachlorido-octahedro-hexamolybdate N,N-dimethylformamide tetrasolvate (1) Atomic displacement parameters (Å^2^)

**Table:**

	*U*^11^	*U*^22^	*U*^33^	*U*^12^	*U*^13^	*U*^23^
Mo1	0.01465 (11)	0.01876 (11)	0.01888 (11)	0.00353 (8)	0.00272 (8)	0.00463 (8)
Mo2	0.01627 (11)	0.01691 (10)	0.01871 (11)	0.00234 (8)	0.00337 (8)	0.00474 (8)
Mo3	0.01646 (12)	0.01852 (11)	0.01703 (11)	0.00379 (8)	0.00436 (8)	0.00307 (8)
Cl1	0.0205 (3)	0.0202 (3)	0.0218 (3)	0.0011 (2)	0.0008 (2)	0.0022 (2)
Cl2	0.0233 (3)	0.0256 (3)	0.0208 (3)	0.0045 (2)	0.0022 (2)	0.0086 (2)
Cl3	0.0231 (3)	0.0211 (3)	0.0272 (3)	0.0086 (2)	0.0041 (2)	0.0046 (2)
Cl4	0.0169 (3)	0.0267 (3)	0.0247 (3)	0.0029 (2)	0.0066 (2)	0.0066 (2)
Cl5	0.0345 (3)	0.0242 (3)	0.0369 (3)	−0.0020 (3)	0.0024 (3)	0.0134 (3)
Cl6	0.0350 (4)	0.0359 (3)	0.0241 (3)	0.0078 (3)	0.0112 (3)	−0.0010 (3)
Cl7	0.0199 (3)	0.0356 (3)	0.0397 (3)	0.0061 (3)	−0.0019 (3)	0.0132 (3)
N1	0.0306 (12)	0.0255 (11)	0.0341 (12)	0.0094 (10)	0.0055 (10)	0.0104 (10)
C1	0.0273 (13)	0.0252 (12)	0.0296 (13)	0.0132 (11)	0.0041 (11)	0.0072 (10)
C2	0.0437 (16)	0.0307 (13)	0.0379 (15)	0.0162 (12)	0.0069 (13)	0.0142 (12)
C3	0.063 (2)	0.0469 (17)	0.0309 (15)	0.0328 (16)	0.0019 (14)	0.0097 (13)
C4	0.0505 (18)	0.0396 (16)	0.0433 (17)	0.0259 (15)	−0.0084 (14)	−0.0073 (13)
C5	0.0372 (15)	0.0263 (13)	0.0383 (15)	0.0145 (11)	0.0117 (12)	0.0089 (11)
C6	0.0412 (17)	0.0277 (14)	0.0495 (18)	0.0115 (12)	0.0063 (14)	−0.0016 (13)
O1	0.0496 (12)	0.0479 (11)	0.0392 (11)	0.0149 (10)	0.0045 (9)	0.0219 (9)
N2	0.0270 (11)	0.0275 (11)	0.0326 (11)	0.0065 (9)	0.0007 (9)	0.0094 (9)
C7	0.0462 (17)	0.0355 (15)	0.0465 (17)	0.0170 (13)	0.0084 (14)	0.0144 (13)
C8	0.0248 (14)	0.0313 (13)	0.0428 (16)	0.0048 (11)	0.0040 (12)	0.0140 (12)
C9	0.0334 (15)	0.0362 (14)	0.0332 (14)	−0.0008 (12)	−0.0040 (12)	0.0062 (12)
O2	0.0359 (11)	0.0345 (10)	0.0614 (13)	0.0134 (9)	0.0149 (10)	0.0192 (9)
N3	0.0309 (12)	0.0407 (12)	0.0301 (11)	0.0169 (10)	0.0066 (10)	0.0120 (10)
C10	0.0391 (16)	0.0390 (15)	0.0470 (17)	0.0112 (13)	0.0145 (13)	0.0197 (13)
C11	0.0527 (19)	0.0527 (18)	0.0427 (17)	0.0301 (15)	0.0149 (15)	0.0142 (14)
C12	0.073 (2)	0.099 (3)	0.0438 (18)	0.053 (2)	0.0248 (17)	0.0425 (19)

### Bis(anilinium) octa-µ3-chlorido-hexachlorido-octahedro-hexamolybdate N,N-dimethylformamide tetrasolvate (1) Geometric parameters (Å, º)

**Table:**

Mo1—Mo2^i^	2.6034 (4)	C3—H3	0.9500
Mo1—Mo2	2.6051 (3)	C3—C4	1.368 (4)
Mo1—Mo3^i^	2.6068 (3)	C4—H4	0.9500
Mo1—Mo3	2.6032 (4)	C4—C6	1.381 (4)
Mo1—Cl1	2.4636 (6)	C5—H5	0.9500
Mo1—Cl2	2.4697 (6)	C5—C6	1.383 (4)
Mo1—Cl3	2.4782 (6)	C6—H6	0.9500
Mo1—Cl4	2.4700 (6)	O1—C8	1.234 (3)
Mo1—Cl7	2.4235 (6)	N2—C7	1.444 (3)
Mo2—Mo3	2.5922 (4)	N2—C8	1.319 (3)
Mo2—Mo3^i^	2.6065 (3)	N2—C9	1.453 (3)
Mo2—Cl1	2.4687 (6)	C7—H7A	0.9800
Mo2—Cl2^i^	2.4772 (6)	C7—H7B	0.9800
Mo2—Cl3^i^	2.4756 (6)	C7—H7C	0.9800
Mo2—Cl4	2.4758 (6)	C8—H8	0.9500
Mo2—Cl5	2.4138 (6)	C9—H9A	0.9800
Mo3—Cl1^i^	2.4754 (6)	C9—H9B	0.9800
Mo3—Cl2^i^	2.4639 (6)	C9—H9C	0.9800
Mo3—Cl3	2.4695 (6)	O2—C10	1.239 (3)
Mo3—Cl4	2.4652 (6)	N3—C10	1.299 (3)
Mo3—Cl6	2.4288 (6)	N3—C11	1.461 (3)
N1—H1A	0.89 (3)	N3—C12	1.449 (3)
N1—H1B	0.91 (3)	C10—H10	0.9500
N1—H1C	0.91 (3)	C11—H11A	0.9800
N1—C1	1.465 (3)	C11—H11B	0.9800
C1—C2	1.376 (3)	C11—H11C	0.9800
C1—C5	1.371 (3)	C12—H12A	0.9800
C2—H2	0.9500	C12—H12B	0.9800
C2—C3	1.378 (4)	C12—H12C	0.9800
			
Mo2^i^—Mo1—Mo2	89.753 (11)	Cl3—Mo3—Mo1^i^	118.261 (15)
Mo2—Mo1—Mo3^i^	60.013 (9)	Cl3—Mo3—Mo2^i^	58.306 (14)
Mo2^i^—Mo1—Mo3^i^	59.675 (10)	Cl3—Mo3—Mo2	118.588 (17)
Mo3—Mo1—Mo2^i^	60.080 (8)	Cl3—Mo3—Cl1^i^	89.67 (2)
Mo3—Mo1—Mo2	59.698 (9)	Cl4—Mo3—Mo1^i^	118.636 (17)
Mo3—Mo1—Mo3^i^	89.786 (11)	Cl4—Mo3—Mo1	58.254 (14)
Cl1—Mo1—Mo2^i^	118.020 (15)	Cl4—Mo3—Mo2	58.556 (14)
Cl1—Mo1—Mo2	58.214 (16)	Cl4—Mo3—Mo2^i^	118.205 (16)
Cl1—Mo1—Mo3	117.899 (15)	Cl4—Mo3—Cl1^i^	175.592 (19)
Cl1—Mo1—Mo3^i^	58.365 (14)	Cl4—Mo3—Cl3	89.93 (2)
Cl1—Mo1—Cl2	89.683 (19)	Cl6—Mo3—Mo1	134.228 (19)
Cl1—Mo1—Cl3	175.251 (19)	Cl6—Mo3—Mo1^i^	135.485 (18)
Cl1—Mo1—Cl4	89.963 (19)	Cl6—Mo3—Mo2^i^	137.228 (18)
Cl2—Mo1—Mo2^i^	58.387 (15)	Cl6—Mo3—Mo2	132.798 (17)
Cl2—Mo1—Mo2	117.999 (16)	Cl6—Mo3—Cl1^i^	94.40 (2)
Cl2—Mo1—Mo3	118.444 (17)	Cl6—Mo3—Cl2^i^	90.84 (2)
Cl2—Mo1—Mo3^i^	57.995 (14)	Cl6—Mo3—Cl3	93.06 (2)
Cl2—Mo1—Cl3	90.38 (2)	Cl6—Mo3—Cl4	90.01 (2)
Cl2—Mo1—Cl4	175.624 (19)	Mo1—Cl1—Mo2	63.764 (15)
Cl3—Mo1—Mo2^i^	58.247 (14)	Mo1—Cl1—Mo3^i^	63.713 (15)
Cl3—Mo1—Mo2	117.771 (17)	Mo2—Cl1—Mo3^i^	63.630 (15)
Cl3—Mo1—Mo3^i^	117.898 (15)	Mo1—Cl2—Mo2^i^	63.507 (16)
Cl3—Mo1—Mo3	58.092 (14)	Mo3^i^—Cl2—Mo1	63.792 (15)
Cl4—Mo1—Mo2^i^	118.144 (16)	Mo3^i^—Cl2—Mo2^i^	63.287 (16)
Cl4—Mo1—Mo2	58.325 (14)	Mo2^i^—Cl3—Mo1	63.408 (14)
Cl4—Mo1—Mo3^i^	118.319 (15)	Mo3—Cl3—Mo1	63.489 (16)
Cl4—Mo1—Mo3	58.077 (16)	Mo3—Cl3—Mo2^i^	63.616 (15)
Cl4—Mo1—Cl3	89.62 (2)	Mo1—Cl4—Mo2	63.569 (15)
Cl7—Mo1—Mo2	135.582 (17)	Mo3—Cl4—Mo1	63.669 (15)
Cl7—Mo1—Mo2^i^	134.650 (17)	Mo3—Cl4—Mo2	63.286 (16)
Cl7—Mo1—Mo3	135.962 (17)	H1A—N1—H1B	101 (2)
Cl7—Mo1—Mo3^i^	134.249 (18)	H1A—N1—H1C	114 (3)
Cl7—Mo1—Cl1	92.00 (2)	H1B—N1—H1C	109 (2)
Cl7—Mo1—Cl2	91.25 (2)	C1—N1—H1A	108.4 (18)
Cl7—Mo1—Cl3	92.75 (2)	C1—N1—H1B	113.6 (18)
Cl7—Mo1—Cl4	93.12 (2)	C1—N1—H1C	111.0 (18)
Mo1^i^—Mo2—Mo1	90.246 (11)	C2—C1—N1	119.1 (2)
Mo1^i^—Mo2—Mo3^i^	59.955 (8)	C5—C1—N1	119.1 (2)
Mo1—Mo2—Mo3^i^	60.026 (9)	C5—C1—C2	121.8 (2)
Mo3—Mo2—Mo1^i^	60.228 (8)	C1—C2—H2	120.6
Mo3—Mo2—Mo1	60.114 (10)	C1—C2—C3	118.8 (3)
Mo3—Mo2—Mo3^i^	90.033 (11)	C3—C2—H2	120.6
Cl1—Mo2—Mo1	58.022 (15)	C2—C3—H3	119.7
Cl1—Mo2—Mo1^i^	118.250 (15)	C4—C3—C2	120.5 (3)
Cl1—Mo2—Mo3^i^	58.310 (15)	C4—C3—H3	119.7
Cl1—Mo2—Mo3	118.122 (15)	C3—C4—H4	120.0
Cl1—Mo2—Cl2^i^	175.448 (18)	C3—C4—C6	120.0 (3)
Cl1—Mo2—Cl3^i^	89.68 (2)	C6—C4—H4	120.0
Cl1—Mo2—Cl4	89.71 (2)	C1—C5—H5	120.7
Cl2^i^—Mo2—Mo1^i^	58.106 (14)	C1—C5—C6	118.6 (3)
Cl2^i^—Mo2—Mo1	118.200 (16)	C6—C5—H5	120.7
Cl2^i^—Mo2—Mo3	58.105 (14)	C4—C6—C5	120.2 (3)
Cl2^i^—Mo2—Mo3^i^	118.038 (16)	C4—C6—H6	119.9
Cl3^i^—Mo2—Mo1^i^	58.346 (16)	C5—C6—H6	119.9
Cl3^i^—Mo2—Mo1	118.097 (16)	C7—N2—C9	117.3 (2)
Cl3^i^—Mo2—Mo3	118.549 (15)	C8—N2—C7	121.2 (2)
Cl3^i^—Mo2—Mo3^i^	58.076 (14)	C8—N2—C9	121.5 (2)
Cl3^i^—Mo2—Cl2^i^	90.26 (2)	N2—C7—H7A	109.5
Cl3^i^—Mo2—Cl4	175.629 (18)	N2—C7—H7B	109.5
Cl4—Mo2—Mo1	58.105 (15)	N2—C7—H7C	109.5
Cl4—Mo2—Mo1^i^	118.367 (16)	H7A—C7—H7B	109.5
Cl4—Mo2—Mo3	58.157 (14)	H7A—C7—H7C	109.5
Cl4—Mo2—Mo3^i^	118.112 (15)	H7B—C7—H7C	109.5
Cl4—Mo2—Cl2^i^	90.00 (2)	O1—C8—N2	124.7 (2)
Cl5—Mo2—Mo1^i^	134.531 (18)	O1—C8—H8	117.6
Cl5—Mo2—Mo1	135.222 (19)	N2—C8—H8	117.6
Cl5—Mo2—Mo3^i^	135.276 (17)	N2—C9—H9A	109.5
Cl5—Mo2—Mo3	134.689 (19)	N2—C9—H9B	109.5
Cl5—Mo2—Cl1	92.63 (2)	N2—C9—H9C	109.5
Cl5—Mo2—Cl2^i^	91.92 (2)	H9A—C9—H9B	109.5
Cl5—Mo2—Cl3^i^	92.04 (2)	H9A—C9—H9C	109.5
Cl5—Mo2—Cl4	92.31 (2)	H9B—C9—H9C	109.5
Mo1—Mo3—Mo1^i^	90.214 (10)	C10—N3—C11	122.7 (2)
Mo1—Mo3—Mo2^i^	59.965 (10)	C10—N3—C12	121.0 (2)
Mo2—Mo3—Mo1^i^	60.098 (8)	C12—N3—C11	116.3 (2)
Mo2^i^—Mo3—Mo1^i^	59.961 (8)	O2—C10—N3	126.1 (3)
Mo2—Mo3—Mo1	60.188 (8)	O2—C10—H10	116.9
Mo2—Mo3—Mo2^i^	89.968 (10)	N3—C10—H10	116.9
Cl1^i^—Mo3—Mo1^i^	57.923 (15)	N3—C11—H11A	109.5
Cl1^i^—Mo3—Mo1	118.008 (15)	N3—C11—H11B	109.5
Cl1^i^—Mo3—Mo2	118.001 (15)	N3—C11—H11C	109.5
Cl1^i^—Mo3—Mo2^i^	58.058 (14)	H11A—C11—H11B	109.5
Cl2^i^—Mo3—Mo1^i^	58.214 (14)	H11A—C11—H11C	109.5
Cl2^i^—Mo3—Mo1	118.777 (16)	H11B—C11—H11C	109.5
Cl2^i^—Mo3—Mo2	58.608 (16)	N3—C12—H12A	109.5
Cl2^i^—Mo3—Mo2^i^	118.165 (15)	N3—C12—H12B	109.5
Cl2^i^—Mo3—Cl1^i^	89.55 (2)	N3—C12—H12C	109.5
Cl2^i^—Mo3—Cl3	176.071 (19)	H12A—C12—H12B	109.5
Cl2^i^—Mo3—Cl4	90.56 (2)	H12A—C12—H12C	109.5
Cl3—Mo3—Mo1	58.419 (15)	H12B—C12—H12C	109.5
			
N1—C1—C2—C3	179.4 (2)	C3—C4—C6—C5	0.4 (4)
N1—C1—C5—C6	−178.9 (2)	C5—C1—C2—C3	−0.5 (4)
C1—C2—C3—C4	0.0 (4)	C7—N2—C8—O1	0.0 (4)
C1—C5—C6—C4	−0.8 (4)	C9—N2—C8—O1	−179.2 (2)
C2—C1—C5—C6	0.9 (4)	C11—N3—C10—O2	−177.1 (3)
C2—C3—C4—C6	0.1 (4)	C12—N3—C10—O2	1.5 (5)

Symmetry code: (i) −*x*+1, −*y*+1, −*z*+1.

### Bis(anilinium) octa-µ3-chlorido-hexachlorido-octahedro-hexamolybdate N,N-dimethylformamide tetrasolvate (1) Hydrogen-bond geometry (Å, º)

**Table:**

*D*—H···*A*	*D*—H	H···*A*	*D*···*A*	*D*—H···*A*
N1—H1*A*···O2^ii^	0.89 (3)	2.01 (3)	2.827 (3)	152 (2)
N1—H1*B*···O2^iii^	0.91 (3)	1.94 (3)	2.833 (3)	168 (3)
N1—H1*C*···O1^iv^	0.91 (3)	1.82 (3)	2.715 (3)	166 (3)

Symmetry codes: (ii) −*x*+1, −*y*+1, −*z*; (iii) *x*−1, *y*, *z*; (iv) *x*, *y*, *z*−1.

### p-Phenylenediammonium octa-µ3-chlorido-hexachlorido-octahedro-hexamolybdate N,N-dimethylformamide hexasolvate (2) Crystal data

**Table:**

(C_6_H_10_N_2_)[Mo_6_Cl_8_Cl_6_]·6C_3_H_7_NO	*Z* = 1
*M**_r_* = 1620.67	*F*(000) = 790
Triclinic, *P*1	*D*_x_ = 2.084 Mg m^−^^3^
*a* = 10.1752 (16) Å	Mo *K*α radiation, λ = 0.71073 Å
*b* = 10.3227 (16) Å	Cell parameters from 4690 reflections
*c* = 13.736 (2) Å	θ = 2.2–25.0°
α = 95.204 (4)°	µ = 2.18 mm^−^^1^
β = 111.483 (4)°	*T* = 200 K
γ = 101.973 (4)°	Needle, yellow
*V* = 1291.1 (3) Å^3^	0.50 × 0.13 × 0.13 mm

### p-Phenylenediammonium octa-µ3-chlorido-hexachlorido-octahedro-hexamolybdate N,N-dimethylformamide hexasolvate (2) Data collection

**Table:**

Bruker SMART X2S benchtop diffractometer	4504 independent reflections
Radiation source: sealed microfocus source, XOS X-beam microfocus source	3666 reflections with *I* > 2σ(*I*)
Graphite monochromator	*R*_int_ = 0.035
Detector resolution: 8.3330 pixels mm^-1^	θ_max_ = 25.0°, θ_min_ = 2.4°
φ and ω scans	*h* = −12→12
Absorption correction: multi-scan (SADABS; Bruker, 2012)	*k* = −12→12
*T*_min_ = 0.552, *T*_max_ = 0.745	*l* = −16→16
12459 measured reflections	

### p-Phenylenediammonium octa-µ3-chlorido-hexachlorido-octahedro-hexamolybdate N,N-dimethylformamide hexasolvate (2) Refinement

**Table:**

Refinement on *F*^2^	Hydrogen site location: mixed
Least-squares matrix: full	H atoms treated by a mixture of independent and constrained refinement
*R*[*F*^2^ > 2σ(*F*^2^)] = 0.026	*w* = 1/[σ^2^(*F*_o_^2^) + (0.0182*P*)^2^ + 0.7035*P*] where *P* = (*F*_o_^2^ + 2*F*_c_^2^)/3
*wR*(*F*^2^) = 0.062	(Δ/σ)_max_ = 0.002
*S* = 1.03	Δρ_max_ = 0.66 e Å^−^^3^
4504 reflections	Δρ_min_ = −0.56 e Å^−^^3^
285 parameters	Extinction correction: SHELXL2018 (Sheldrick, 2015), Fc^*^=kFc[1+0.001xFc^2^λ^3^/sin(2θ)]^-1/4^
144 restraints	Extinction coefficient: 0.0019 (2)
Primary atom site location: structure-invariant direct methods	

### p-Phenylenediammonium octa-µ3-chlorido-hexachlorido-octahedro-hexamolybdate N,N-dimethylformamide hexasolvate (2) Special details

**Table:**

Geometry. All esds (except the esd in the dihedral angle between two l.s. planes) are estimated using the full covariance matrix. The cell esds are taken into account individually in the estimation of esds in distances, angles and torsion angles; correlations between esds in cell parameters are only used when they are defined by crystal symmetry. An approximate (isotropic) treatment of cell esds is used for estimating esds involving l.s. planes.

### p-Phenylenediammonium octa-µ3-chlorido-hexachlorido-octahedro-hexamolybdate N,N-dimethylformamide hexasolvate (2) Fractional atomic coordinates and isotropic or equivalent isotropic displacement parameters (Å^2^)

**Table:**

	*x*	*y*	*z*	*U*_iso_*/*U*_eq_	Occ. (<1)
Mo1	0.38576 (3)	0.32569 (3)	0.46504 (2)	0.01984 (9)	
Mo2	0.38226 (3)	0.53457 (3)	0.37203 (2)	0.01942 (9)	
Mo3	0.61719 (3)	0.44772 (3)	0.43759 (2)	0.01961 (9)	
Cl1	0.39098 (9)	0.31721 (8)	0.28606 (6)	0.02470 (19)	
Cl2	0.16889 (8)	0.41769 (8)	0.40411 (7)	0.02485 (19)	
Cl3	0.61432 (9)	0.25211 (8)	0.52889 (7)	0.02446 (19)	
Cl4	0.39296 (9)	0.35261 (8)	0.64765 (6)	0.02436 (19)	
Cl5	0.23637 (10)	0.09538 (8)	0.42076 (7)	0.0329 (2)	
Cl6	0.22927 (9)	0.58540 (9)	0.20362 (7)	0.0303 (2)	
Cl7	0.77350 (10)	0.38668 (9)	0.35511 (7)	0.0340 (2)	
N1	0.6572 (4)	0.7709 (3)	0.0311 (3)	0.0349 (8)	
H1A	0.593 (4)	0.816 (4)	−0.008 (3)	0.052*	
H1B	0.735 (3)	0.773 (4)	0.010 (3)	0.052*	
H1C	0.702 (4)	0.814 (4)	0.1013 (17)	0.052*	
C1	0.5771 (4)	0.6310 (4)	0.0161 (3)	0.0300 (8)	
C2	0.4838 (4)	0.5982 (4)	0.0660 (3)	0.0346 (10)	0.918 (4)
H2	0.473071	0.666286	0.111672	0.041*	0.918 (4)
C2A	0.614 (4)	0.562 (3)	0.098 (2)	0.0346 (10)	0.082 (4)
H2A	0.690135	0.603414	0.164923	0.041*	0.082 (4)
C3	0.5946 (4)	0.5334 (4)	−0.0500 (3)	0.0368 (11)	0.918 (4)
H3	0.659871	0.556614	−0.084221	0.044*	0.918 (4)
C3A	0.465 (2)	0.570 (3)	−0.0792 (18)	0.0368 (11)	0.082 (4)
H3A	0.440687	0.620672	−0.134626	0.044*	0.082 (4)
O1	0.2262 (3)	0.1030 (3)	0.7614 (2)	0.0397 (7)	
N2	0.0590 (3)	0.1987 (3)	0.6536 (2)	0.0345 (7)	
C4	−0.0193 (4)	0.2197 (4)	0.5464 (3)	0.0471 (11)	
H4A	0.012128	0.173444	0.496723	0.071*	
H4B	0.001881	0.316351	0.544827	0.071*	
H4C	−0.124667	0.183480	0.525552	0.071*	
C5	0.0344 (6)	0.2671 (6)	0.7385 (4)	0.089 (2)	
H5A	0.105377	0.355397	0.767114	0.133*	
H5B	0.045962	0.213536	0.795020	0.133*	
H5C	−0.064912	0.279168	0.711359	0.133*	
C6	0.1511 (4)	0.1229 (3)	0.6738 (3)	0.0310 (9)	
H6	0.160825	0.078393	0.614092	0.037*	
O2	0.4557 (3)	−0.1235 (3)	0.9024 (2)	0.0528 (8)	
N3	0.5360 (3)	−0.0093 (3)	0.7928 (2)	0.0365 (8)	
C7	0.6841 (5)	0.0426 (5)	0.8704 (4)	0.0737 (16)	
H7A	0.742668	−0.019070	0.863107	0.111*	
H7B	0.684310	0.050996	0.942109	0.111*	
H7C	0.726072	0.131344	0.858773	0.111*	
C8	0.5004 (5)	0.0294 (4)	0.6892 (3)	0.0521 (12)	
H8A	0.516051	0.127409	0.697400	0.078*	
H8B	0.397800	−0.014548	0.643792	0.078*	
H8C	0.563372	0.001622	0.656359	0.078*	
C9	0.4359 (4)	−0.0854 (4)	0.8172 (4)	0.0401 (10)	
H9	0.339326	−0.113538	0.763903	0.048*	
O3	1.1120 (3)	0.2000 (3)	0.0265 (2)	0.0531 (8)	
N4	0.9538 (3)	0.2480 (3)	0.0950 (2)	0.0348 (7)	
C10	0.8672 (5)	0.3384 (4)	0.1084 (4)	0.0542 (12)	
H10A	0.910401	0.386797	0.182173	0.081*	
H10B	0.767006	0.286161	0.091710	0.081*	
H10C	0.865666	0.403223	0.060362	0.081*	
C11	0.9567 (5)	0.1356 (4)	0.1512 (3)	0.0513 (11)	
H11A	0.986243	0.066079	0.116745	0.077*	
H11B	0.859138	0.097880	0.149607	0.077*	
H11C	1.026857	0.166773	0.225219	0.077*	
C12	1.0327 (4)	0.2695 (4)	0.0379 (3)	0.0415 (10)	
H12	1.027845	0.344630	0.002820	0.050*	

### p-Phenylenediammonium octa-µ3-chlorido-hexachlorido-octahedro-hexamolybdate N,N-dimethylformamide hexasolvate (2) Atomic displacement parameters (Å^2^)

**Table:**

	*U*^11^	*U*^22^	*U*^33^	*U*^12^	*U*^13^	*U*^23^
Mo1	0.01844 (16)	0.01776 (16)	0.02281 (18)	0.00192 (12)	0.00933 (13)	0.00253 (12)
Mo2	0.01778 (16)	0.01982 (16)	0.02082 (18)	0.00380 (12)	0.00849 (13)	0.00326 (12)
Mo3	0.01810 (16)	0.01988 (16)	0.02220 (18)	0.00434 (12)	0.01006 (13)	0.00274 (12)
Cl1	0.0253 (4)	0.0231 (4)	0.0234 (5)	0.0034 (3)	0.0097 (4)	−0.0003 (3)
Cl2	0.0174 (4)	0.0274 (4)	0.0279 (5)	0.0026 (3)	0.0088 (4)	0.0041 (4)
Cl3	0.0252 (4)	0.0212 (4)	0.0287 (5)	0.0079 (3)	0.0115 (4)	0.0047 (4)
Cl4	0.0249 (4)	0.0248 (4)	0.0264 (5)	0.0041 (3)	0.0142 (4)	0.0067 (4)
Cl5	0.0336 (5)	0.0222 (4)	0.0385 (5)	−0.0019 (4)	0.0151 (4)	0.0018 (4)
Cl6	0.0290 (5)	0.0361 (5)	0.0254 (5)	0.0108 (4)	0.0087 (4)	0.0072 (4)
Cl7	0.0331 (5)	0.0407 (5)	0.0373 (5)	0.0137 (4)	0.0223 (4)	0.0051 (4)
N1	0.0345 (19)	0.039 (2)	0.0338 (19)	0.0056 (16)	0.0181 (16)	0.0081 (16)
C1	0.028 (2)	0.036 (2)	0.029 (2)	0.0097 (17)	0.0132 (17)	0.0136 (17)
C2	0.039 (2)	0.038 (2)	0.032 (2)	0.015 (2)	0.018 (2)	0.0055 (19)
C2A	0.039 (2)	0.038 (2)	0.032 (2)	0.015 (2)	0.018 (2)	0.0055 (19)
C3	0.035 (2)	0.049 (3)	0.033 (2)	0.010 (2)	0.021 (2)	0.012 (2)
C3A	0.035 (2)	0.049 (3)	0.033 (2)	0.010 (2)	0.021 (2)	0.012 (2)
O1	0.0426 (16)	0.0405 (16)	0.0385 (17)	0.0171 (13)	0.0145 (14)	0.0111 (13)
N2	0.0296 (17)	0.0395 (19)	0.040 (2)	0.0109 (15)	0.0191 (15)	0.0086 (15)
C4	0.030 (2)	0.060 (3)	0.054 (3)	0.013 (2)	0.015 (2)	0.027 (2)
C5	0.109 (5)	0.126 (5)	0.065 (4)	0.083 (4)	0.045 (3)	0.016 (4)
C6	0.032 (2)	0.0247 (19)	0.039 (2)	0.0027 (17)	0.0197 (19)	0.0024 (17)
O2	0.0527 (19)	0.0526 (19)	0.052 (2)	0.0122 (15)	0.0172 (16)	0.0249 (16)
N3	0.0329 (18)	0.0305 (18)	0.040 (2)	0.0080 (15)	0.0073 (15)	0.0089 (15)
C7	0.048 (3)	0.060 (3)	0.082 (4)	−0.008 (3)	−0.001 (3)	0.027 (3)
C8	0.076 (3)	0.054 (3)	0.041 (3)	0.032 (3)	0.029 (2)	0.016 (2)
C9	0.033 (2)	0.027 (2)	0.051 (3)	0.0096 (18)	0.007 (2)	0.003 (2)
O3	0.0490 (18)	0.0476 (18)	0.071 (2)	0.0059 (15)	0.0388 (17)	0.0033 (16)
N4	0.0308 (17)	0.0370 (19)	0.0326 (19)	0.0017 (15)	0.0122 (15)	0.0045 (15)
C10	0.047 (3)	0.057 (3)	0.064 (3)	0.012 (2)	0.028 (2)	0.008 (2)
C11	0.051 (3)	0.055 (3)	0.048 (3)	0.009 (2)	0.021 (2)	0.020 (2)
C12	0.039 (2)	0.040 (2)	0.037 (2)	−0.004 (2)	0.014 (2)	0.0043 (19)

### p-Phenylenediammonium octa-µ3-chlorido-hexachlorido-octahedro-hexamolybdate N,N-dimethylformamide hexasolvate (2) Geometric parameters (Å, º)

**Table:**

Mo1—Mo2^i^	2.6065 (5)	C3A—H3A	0.9500
Mo1—Mo2	2.6040 (5)	O1—C6	1.229 (4)
Mo1—Mo3	2.6039 (5)	N2—C4	1.456 (5)
Mo1—Mo3^i^	2.5984 (5)	N2—C5	1.435 (5)
Mo1—Cl1	2.4724 (9)	N2—C6	1.312 (4)
Mo1—Cl2	2.4764 (9)	C4—H4A	0.9800
Mo1—Cl3	2.4727 (9)	C4—H4B	0.9800
Mo1—Cl4	2.4708 (9)	C4—H4C	0.9800
Mo1—Cl5	2.4277 (9)	C5—H5A	0.9800
Mo2—Mo3^i^	2.6027 (5)	C5—H5B	0.9800
Mo2—Mo3	2.6055 (5)	C5—H5C	0.9800
Mo2—Cl1	2.4729 (9)	C6—H6	0.9500
Mo2—Cl2	2.4644 (9)	O2—C9	1.228 (5)
Mo2—Cl3^i^	2.4682 (9)	N3—C7	1.442 (5)
Mo2—Cl4^i^	2.4629 (9)	N3—C8	1.450 (5)
Mo2—Cl6	2.4436 (9)	N3—C9	1.316 (5)
Mo3—Cl1	2.4781 (9)	C7—H7A	0.9800
Mo3—Cl2^i^	2.4751 (9)	C7—H7B	0.9800
Mo3—Cl3	2.4724 (9)	C7—H7C	0.9800
Mo3—Cl4^i^	2.4632 (9)	C8—H8A	0.9800
Mo3—Cl7	2.4116 (9)	C8—H8B	0.9800
N1—H1A	0.922 (18)	C8—H8C	0.9800
N1—H1B	0.927 (18)	C9—H9	0.9500
N1—H1C	0.924 (18)	O3—C12	1.227 (5)
N1—C1	1.458 (5)	N4—C10	1.453 (5)
C1—C2	1.367 (5)	N4—C11	1.450 (5)
C1—C2A	1.361 (16)	N4—C12	1.314 (5)
C1—C3	1.377 (5)	C10—H10A	0.9800
C1—C3A	1.366 (16)	C10—H10B	0.9800
C2—H2	0.9500	C10—H10C	0.9800
C2—C3^ii^	1.378 (5)	C11—H11A	0.9800
C2A—H2A	0.9500	C11—H11B	0.9800
C2A—C3A^ii^	1.380 (15)	C11—H11C	0.9800
C3—H3	0.9500	C12—H12	0.9500
			
Mo2—Mo1—Mo2^i^	90.126 (16)	Cl4^i^—Mo3—Cl3	175.60 (3)
Mo3—Mo1—Mo2^i^	59.936 (14)	Cl7—Mo3—Mo1	136.77 (3)
Mo3—Mo1—Mo2	60.038 (12)	Cl7—Mo3—Mo1^i^	133.17 (3)
Mo3^i^—Mo1—Mo2^i^	60.074 (13)	Cl7—Mo3—Mo2^i^	135.56 (3)
Mo3^i^—Mo1—Mo2	60.036 (15)	Cl7—Mo3—Mo2	134.21 (3)
Mo3^i^—Mo1—Mo3	89.946 (14)	Cl7—Mo3—Cl1	92.98 (3)
Cl1—Mo1—Mo2^i^	118.30 (2)	Cl7—Mo3—Cl2^i^	91.41 (3)
Cl1—Mo1—Mo2	58.24 (2)	Cl7—Mo3—Cl3	93.96 (3)
Cl1—Mo1—Mo3^i^	118.26 (2)	Cl7—Mo3—Cl4^i^	90.44 (3)
Cl1—Mo1—Mo3	58.37 (2)	Mo1—Cl1—Mo2	63.55 (2)
Cl1—Mo1—Cl2	89.61 (3)	Mo1—Cl1—Mo3	63.47 (2)
Cl1—Mo1—Cl3	90.01 (3)	Mo2—Cl1—Mo3	63.50 (2)
Cl2—Mo1—Mo2	57.97 (2)	Mo2—Cl2—Mo1	63.61 (2)
Cl2—Mo1—Mo2^i^	118.37 (2)	Mo2—Cl2—Mo3^i^	63.59 (2)
Cl2—Mo1—Mo3^i^	58.32 (2)	Mo3^i^—Cl2—Mo1	63.31 (2)
Cl2—Mo1—Mo3	117.99 (2)	Mo2^i^—Cl3—Mo1	63.68 (2)
Cl3—Mo1—Mo2^i^	58.08 (2)	Mo2^i^—Cl3—Mo3	63.58 (2)
Cl3—Mo1—Mo2	118.24 (2)	Mo3—Cl3—Mo1	63.55 (2)
Cl3—Mo1—Mo3^i^	118.13 (2)	Mo2^i^—Cl4—Mo1	63.78 (2)
Cl3—Mo1—Mo3	58.22 (2)	Mo2^i^—Cl4—Mo3^i^	63.86 (2)
Cl3—Mo1—Cl2	175.50 (3)	Mo3^i^—Cl4—Mo1	63.56 (2)
Cl4—Mo1—Mo2^i^	57.96 (2)	H1A—N1—H1B	112 (4)
Cl4—Mo1—Mo2	118.09 (2)	H1A—N1—H1C	110 (4)
Cl4—Mo1—Mo3	117.88 (2)	H1B—N1—H1C	103 (3)
Cl4—Mo1—Mo3^i^	58.08 (2)	C1—N1—H1A	108 (3)
Cl4—Mo1—Cl1	175.46 (3)	C1—N1—H1B	109 (3)
Cl4—Mo1—Cl2	90.28 (3)	C1—N1—H1C	114 (3)
Cl4—Mo1—Cl3	89.74 (3)	C2—C1—N1	119.9 (3)
Cl5—Mo1—Mo2^i^	134.38 (3)	C2—C1—C3	120.7 (4)
Cl5—Mo1—Mo2	135.49 (3)	C2A—C1—N1	119.6 (15)
Cl5—Mo1—Mo3	135.18 (2)	C2A—C1—C3A	120.3 (10)
Cl5—Mo1—Mo3^i^	134.87 (3)	C3—C1—N1	119.4 (3)
Cl5—Mo1—Cl1	92.58 (3)	C3A—C1—N1	120.1 (16)
Cl5—Mo1—Cl2	92.57 (3)	C1—C2—H2	119.9
Cl5—Mo1—Cl3	91.93 (3)	C1—C2—C3^ii^	120.1 (4)
Cl5—Mo1—Cl4	91.96 (3)	C3^ii^—C2—H2	119.9
Mo1—Mo2—Mo1^i^	89.873 (16)	C1—C2A—H2A	121.4
Mo1—Mo2—Mo3	59.978 (12)	C1—C2A—C3A^ii^	117 (3)
Mo3^i^—Mo2—Mo1^i^	59.981 (14)	C3A^ii^—C2A—H2A	121.4
Mo3^i^—Mo2—Mo1	59.875 (13)	C1—C3—C2^ii^	119.2 (4)
Mo3—Mo2—Mo1^i^	59.809 (14)	C1—C3—H3	120.4
Mo3^i^—Mo2—Mo3	89.819 (14)	C2^ii^—C3—H3	120.4
Cl1—Mo2—Mo1^i^	118.14 (2)	C1—C3A—C2A^ii^	122 (3)
Cl1—Mo2—Mo1	58.22 (2)	C1—C3A—H3A	118.8
Cl1—Mo2—Mo3^i^	118.08 (2)	C2A^ii^—C3A—H3A	118.8
Cl1—Mo2—Mo3	58.34 (2)	C5—N2—C4	117.3 (3)
Cl2—Mo2—Mo1^i^	118.37 (2)	C6—N2—C4	122.3 (3)
Cl2—Mo2—Mo1	58.42 (2)	C6—N2—C5	120.4 (3)
Cl2—Mo2—Mo3	118.38 (2)	N2—C4—H4A	109.5
Cl2—Mo2—Mo3^i^	58.40 (2)	N2—C4—H4B	109.5
Cl2—Mo2—Cl1	89.88 (3)	N2—C4—H4C	109.5
Cl2—Mo2—Cl3^i^	89.95 (3)	H4A—C4—H4B	109.5
Cl3^i^—Mo2—Mo1^i^	58.25 (2)	H4A—C4—H4C	109.5
Cl3^i^—Mo2—Mo1	118.15 (2)	H4B—C4—H4C	109.5
Cl3^i^—Mo2—Mo3^i^	58.29 (2)	N2—C5—H5A	109.5
Cl3^i^—Mo2—Mo3	118.04 (2)	N2—C5—H5B	109.5
Cl3^i^—Mo2—Cl1	175.56 (3)	N2—C5—H5C	109.5
Cl4^i^—Mo2—Mo1	118.04 (2)	H5A—C5—H5B	109.5
Cl4^i^—Mo2—Mo1^i^	58.26 (2)	H5A—C5—H5C	109.5
Cl4^i^—Mo2—Mo3	58.07 (2)	H5B—C5—H5C	109.5
Cl4^i^—Mo2—Mo3^i^	118.22 (2)	O1—C6—N2	127.2 (4)
Cl4^i^—Mo2—Cl1	89.81 (3)	O1—C6—H6	116.4
Cl4^i^—Mo2—Cl2	175.77 (3)	N2—C6—H6	116.4
Cl4^i^—Mo2—Cl3^i^	90.03 (3)	C7—N3—C8	117.8 (4)
Cl6—Mo2—Mo1^i^	133.87 (3)	C9—N3—C7	120.7 (4)
Cl6—Mo2—Mo1	136.26 (3)	C9—N3—C8	121.5 (4)
Cl6—Mo2—Mo3^i^	134.96 (3)	N3—C7—H7A	109.5
Cl6—Mo2—Mo3	135.19 (2)	N3—C7—H7B	109.5
Cl6—Mo2—Cl1	93.14 (3)	N3—C7—H7C	109.5
Cl6—Mo2—Cl2	92.74 (3)	H7A—C7—H7B	109.5
Cl6—Mo2—Cl3^i^	91.30 (3)	H7A—C7—H7C	109.5
Cl6—Mo2—Cl4^i^	91.49 (3)	H7B—C7—H7C	109.5
Mo1^i^—Mo3—Mo1	90.053 (14)	N3—C8—H8A	109.5
Mo1^i^—Mo3—Mo2^i^	60.086 (12)	N3—C8—H8B	109.5
Mo1—Mo3—Mo2	59.984 (14)	N3—C8—H8C	109.5
Mo1^i^—Mo3—Mo2	60.116 (12)	H8A—C8—H8B	109.5
Mo2^i^—Mo3—Mo1	60.082 (11)	H8A—C8—H8C	109.5
Mo2^i^—Mo3—Mo2	90.179 (13)	H8B—C8—H8C	109.5
Cl1—Mo3—Mo1^i^	118.25 (2)	O2—C9—N3	126.1 (4)
Cl1—Mo3—Mo1	58.16 (2)	O2—C9—H9	116.9
Cl1—Mo3—Mo2	58.15 (2)	N3—C9—H9	116.9
Cl1—Mo3—Mo2^i^	118.23 (2)	C11—N4—C10	117.9 (3)
Cl2^i^—Mo3—Mo1	118.07 (2)	C12—N4—C10	121.7 (3)
Cl2^i^—Mo3—Mo1^i^	58.37 (2)	C12—N4—C11	120.3 (4)
Cl2^i^—Mo3—Mo2	118.46 (2)	N4—C10—H10A	109.5
Cl2^i^—Mo3—Mo2^i^	58.00 (2)	N4—C10—H10B	109.5
Cl2^i^—Mo3—Cl1	175.60 (3)	N4—C10—H10C	109.5
Cl3—Mo3—Mo1	58.23 (2)	H10A—C10—H10B	109.5
Cl3—Mo3—Mo1^i^	118.20 (2)	H10A—C10—H10C	109.5
Cl3—Mo3—Mo2	118.20 (2)	H10B—C10—H10C	109.5
Cl3—Mo3—Mo2^i^	58.13 (2)	N4—C11—H11A	109.5
Cl3—Mo3—Cl1	89.89 (3)	N4—C11—H11B	109.5
Cl3—Mo3—Cl2^i^	89.61 (3)	N4—C11—H11C	109.5
Cl4^i^—Mo3—Mo1	118.03 (2)	H11A—C11—H11B	109.5
Cl4^i^—Mo3—Mo1^i^	58.36 (2)	H11A—C11—H11C	109.5
Cl4^i^—Mo3—Mo2^i^	118.43 (2)	H11B—C11—H11C	109.5
Cl4^i^—Mo3—Mo2	58.06 (2)	O3—C12—N4	125.0 (4)
Cl4^i^—Mo3—Cl1	89.68 (3)	O3—C12—H12	117.5
Cl4^i^—Mo3—Cl2^i^	90.48 (3)	N4—C12—H12	117.5
			
N1—C1—C2—C3^ii^	178.5 (3)	C3A—C1—C2A—C3A^ii^	1.0 (17)
N1—C1—C2A—C3A^ii^	−179.7 (9)	C4—N2—C6—O1	177.0 (4)
N1—C1—C3—C2^ii^	−178.5 (3)	C5—N2—C6—O1	−1.6 (6)
N1—C1—C3A—C2A^ii^	179.7 (9)	C7—N3—C9—O2	−2.6 (6)
C2—C1—C3—C2^ii^	0.3 (6)	C8—N3—C9—O2	179.8 (4)
C2A—C1—C3A—C2A^ii^	−1.1 (18)	C10—N4—C12—O3	177.3 (4)
C3—C1—C2—C3^ii^	−0.3 (6)	C11—N4—C12—O3	0.4 (6)

Symmetry codes: (i) −*x*+1, −*y*+1, −*z*+1; (ii) −*x*+1, −*y*+1, −*z*.

### p-Phenylenediammonium octa-µ3-chlorido-hexachlorido-octahedro-hexamolybdate N,N-dimethylformamide hexasolvate (2) Hydrogen-bond geometry (Å, º)

**Table:**

*D*—H···*A*	*D*—H	H···*A*	*D*···*A*	*D*—H···*A*
N1—H1*A*···O2^iii^	0.92 (2)	1.76 (2)	2.672 (4)	171 (4)
N1—H1*B*···O3^iv^	0.93 (2)	1.79 (2)	2.710 (4)	173 (4)
N1—H1*C*···O1^i^	0.92 (2)	1.81 (2)	2.727 (4)	175 (4)

Symmetry codes: (i) −*x*+1, −*y*+1, −*z*+1; (iii) *x*, *y*+1, *z*−1; (iv) −*x*+2, −*y*+1, −*z*.

### N,N'-(1,4-Phenylene)bis(propan-2-iminium) octa-µ3-chlorido-hexachlorido-octahedro-hexamolybdate acetone trisolvate (3) Crystal data

**Table:**

(C_12_H_18_N_2_)[Mo_6_Cl_8_Cl_6_]·3C_3_H_6_O	*Z* = 1
*M**_r_* = 1436.46	*F*(000) = 690
Triclinic, *P*1	*D*_x_ = 2.257 Mg m^−^^3^
*a* = 9.451 (2) Å	Mo *K*α radiation, λ = 0.71073 Å
*b* = 11.236 (3) Å	Cell parameters from 5309 reflections
*c* = 11.712 (3) Å	θ = 2.2–25.1°
α = 64.933 (6)°	µ = 2.64 mm^−^^1^
β = 71.174 (6)°	*T* = 200 K
γ = 75.440 (6)°	Block, clear light orange
*V* = 1056.7 (5) Å^3^	0.55 × 0.33 × 0.20 mm

### N,N'-(1,4-Phenylene)bis(propan-2-iminium) octa-µ3-chlorido-hexachlorido-octahedro-hexamolybdate acetone trisolvate (3) Data collection

**Table:**

Bruker SMART X2S benchtop diffractometer	3692 independent reflections
Radiation source: sealed microfocus source, XOS X-beam microfocus source	3220 reflections with *I* > 2σ(*I*)
Graphite monochromator	*R*_int_ = 0.030
Detector resolution: 8.3330 pixels mm^-1^	θ_max_ = 25.2°, θ_min_ = 2.6°
φ and ω scans	*h* = −11→11
Absorption correction: multi-scan (SADABS; Bruker, 2012)	*k* = −13→13
*T*_min_ = 0.490, *T*_max_ = 0.745	*l* = −13→13
10036 measured reflections	

### N,N'-(1,4-Phenylene)bis(propan-2-iminium) octa-µ3-chlorido-hexachlorido-octahedro-hexamolybdate acetone trisolvate (3) Refinement

**Table:**

Refinement on *F*^2^	Primary atom site location: heavy-atom method
Least-squares matrix: full	Hydrogen site location: mixed
*R*[*F*^2^ > 2σ(*F*^2^)] = 0.025	H atoms treated by a mixture of independent and constrained refinement
*wR*(*F*^2^) = 0.068	*w* = 1/[σ^2^(*F*_o_^2^) + (0.0347*P*)^2^ + 0.3989*P*] where *P* = (*F*_o_^2^ + 2*F*_c_^2^)/3
*S* = 1.05	(Δ/σ)_max_ = 0.001
3692 reflections	Δρ_max_ = 0.96 e Å^−^^3^
235 parameters	Δρ_min_ = −0.82 e Å^−^^3^
13 restraints	

### N,N'-(1,4-Phenylene)bis(propan-2-iminium) octa-µ3-chlorido-hexachlorido-octahedro-hexamolybdate acetone trisolvate (3) Special details

**Table:**

Geometry. All esds (except the esd in the dihedral angle between two l.s. planes) are estimated using the full covariance matrix. The cell esds are taken into account individually in the estimation of esds in distances, angles and torsion angles; correlations between esds in cell parameters are only used when they are defined by crystal symmetry. An approximate (isotropic) treatment of cell esds is used for estimating esds involving l.s. planes.

### N,N'-(1,4-Phenylene)bis(propan-2-iminium) octa-µ3-chlorido-hexachlorido-octahedro-hexamolybdate acetone trisolvate (3) Fractional atomic coordinates and isotropic or equivalent isotropic displacement parameters (Å^2^)

**Table:**

	*x*	*y*	*z*	*U*_iso_*/*U*_eq_	Occ. (<1)
Mo1	0.54044 (3)	0.46936 (2)	0.65449 (2)	0.01669 (9)	
Mo2	0.46251 (3)	0.32776 (2)	0.56169 (2)	0.01681 (9)	
Mo3	0.29965 (3)	0.55064 (2)	0.56544 (2)	0.01718 (9)	
Cl1	0.41212 (10)	0.10003 (8)	0.64118 (8)	0.0324 (2)	
Cl2	0.76390 (8)	0.58538 (7)	0.52537 (7)	0.02273 (17)	
Cl3	0.69229 (9)	0.25958 (7)	0.64379 (7)	0.02413 (18)	
Cl4	0.59212 (10)	0.43277 (8)	0.85707 (7)	0.0303 (2)	
Cl5	0.38283 (9)	0.68264 (7)	0.65005 (7)	0.02236 (17)	
Cl6	0.31409 (9)	0.35417 (8)	0.76707 (7)	0.02377 (18)	
Cl7	0.03772 (9)	0.61748 (9)	0.65222 (9)	0.0360 (2)	
N1	0.2062 (3)	0.0615 (3)	0.2565 (3)	0.0242 (6)	
H1	0.196 (4)	0.021 (3)	0.211 (3)	0.036*	
C1	0.4140 (4)	0.1369 (4)	0.0787 (3)	0.0382 (9)	
H1A	0.431717	0.228439	0.022574	0.057*	
H1B	0.366916	0.102550	0.037660	0.057*	
H1C	0.510355	0.081919	0.091601	0.057*	
C2	0.3131 (4)	0.1337 (3)	0.2058 (3)	0.0265 (7)	
C3	0.3406 (4)	0.2109 (3)	0.2704 (4)	0.0370 (9)	
H3A	0.424810	0.163487	0.311157	0.056*	
H3B	0.249770	0.222227	0.337134	0.056*	
H3C	0.365572	0.298048	0.205817	0.056*	
C4	0.1507 (4)	−0.0037 (3)	0.4928 (3)	0.0281 (7)	
H4	0.253881	−0.006002	0.486934	0.034*	
C5	0.1019 (4)	0.0328 (3)	0.3819 (3)	0.0227 (7)	
C6	−0.0482 (4)	0.0368 (3)	0.3885 (3)	0.0274 (7)	
H6	−0.079966	0.062241	0.311317	0.033*	
O1	0.1737 (3)	0.9573 (3)	0.0897 (2)	0.0440 (7)	
C7	0.0245 (5)	0.7839 (4)	0.1942 (4)	0.0502 (11)	
H7A	0.012696	0.802221	0.271696	0.075*	
H7B	0.067673	0.691137	0.209401	0.075*	
H7C	−0.074289	0.800190	0.175911	0.075*	
C8	0.1262 (4)	0.8717 (4)	0.0818 (4)	0.0345 (8)	
C9	0.1705 (5)	0.8501 (4)	−0.0432 (4)	0.0468 (10)	
H9A	0.205823	0.930184	−0.115350	0.070*	
H9B	0.083109	0.830392	−0.056688	0.070*	
H9C	0.251527	0.775370	−0.039653	0.070*	
O2	0.0767 (6)	0.3001 (5)	0.0578 (5)	0.0461 (13)	0.5
C10	0.0375 (9)	0.4167 (7)	0.0246 (6)	0.0351 (17)	0.5
C11	0.143 (2)	0.512 (2)	−0.011 (2)	0.049 (5)	0.5
H11A	0.117815	0.548863	0.056809	0.073*	0.5
H11B	0.134909	0.584134	−0.094095	0.073*	0.5
H11C	0.246860	0.466249	−0.018685	0.073*	0.5
C12	−0.1156 (17)	0.473 (2)	0.006 (2)	0.044 (5)	0.5
H12A	−0.114886	0.495968	−0.085108	0.067*	0.5
H12B	−0.148442	0.553655	0.026059	0.067*	0.5
H12C	−0.185388	0.408443	0.063119	0.067*	0.5

### N,N'-(1,4-Phenylene)bis(propan-2-iminium) octa-µ3-chlorido-hexachlorido-octahedro-hexamolybdate acetone trisolvate (3) Atomic displacement parameters (Å^2^)

**Table:**

	*U*^11^	*U*^22^	*U*^33^	*U*^12^	*U*^13^	*U*^23^
Mo1	0.01921 (17)	0.01543 (15)	0.01644 (15)	−0.00245 (12)	−0.00747 (11)	−0.00459 (11)
Mo2	0.01936 (17)	0.01344 (15)	0.01826 (15)	−0.00251 (11)	−0.00714 (11)	−0.00450 (11)
Mo3	0.01548 (17)	0.01715 (15)	0.01989 (15)	−0.00117 (11)	−0.00588 (11)	−0.00728 (12)
Cl1	0.0385 (5)	0.0179 (4)	0.0390 (5)	−0.0077 (4)	−0.0110 (4)	−0.0057 (3)
Cl2	0.0208 (4)	0.0243 (4)	0.0272 (4)	−0.0053 (3)	−0.0092 (3)	−0.0098 (3)
Cl3	0.0278 (4)	0.0172 (4)	0.0286 (4)	0.0030 (3)	−0.0158 (3)	−0.0065 (3)
Cl4	0.0429 (5)	0.0301 (4)	0.0223 (4)	−0.0098 (4)	−0.0159 (4)	−0.0053 (3)
Cl5	0.0248 (4)	0.0208 (4)	0.0247 (4)	−0.0008 (3)	−0.0075 (3)	−0.0118 (3)
Cl6	0.0266 (4)	0.0245 (4)	0.0186 (4)	−0.0084 (3)	−0.0029 (3)	−0.0057 (3)
Cl7	0.0189 (4)	0.0453 (5)	0.0457 (5)	0.0015 (4)	−0.0055 (4)	−0.0237 (4)
N1	0.0263 (16)	0.0219 (14)	0.0257 (14)	−0.0046 (12)	−0.0068 (12)	−0.0090 (11)
C1	0.040 (2)	0.036 (2)	0.0310 (19)	−0.0163 (18)	−0.0023 (16)	−0.0037 (16)
C2	0.0241 (19)	0.0158 (16)	0.0354 (18)	0.0018 (14)	−0.0126 (15)	−0.0045 (14)
C3	0.037 (2)	0.0235 (18)	0.054 (2)	−0.0030 (16)	−0.0155 (18)	−0.0152 (17)
C4	0.0213 (18)	0.0312 (18)	0.0346 (18)	−0.0018 (15)	−0.0099 (15)	−0.0136 (15)
C5	0.0251 (19)	0.0163 (15)	0.0275 (16)	0.0008 (13)	−0.0074 (14)	−0.0102 (13)
C6	0.028 (2)	0.0311 (18)	0.0265 (17)	−0.0021 (15)	−0.0113 (14)	−0.0115 (15)
O1	0.0565 (18)	0.0433 (16)	0.0429 (15)	−0.0198 (14)	−0.0152 (13)	−0.0166 (13)
C7	0.038 (2)	0.047 (2)	0.063 (3)	−0.012 (2)	−0.013 (2)	−0.014 (2)
C8	0.031 (2)	0.0322 (19)	0.043 (2)	0.0004 (16)	−0.0204 (17)	−0.0105 (16)
C9	0.057 (3)	0.043 (2)	0.050 (2)	−0.001 (2)	−0.028 (2)	−0.018 (2)
O2	0.051 (4)	0.037 (3)	0.052 (3)	−0.007 (3)	−0.016 (3)	−0.015 (3)
C10	0.046 (5)	0.038 (5)	0.020 (3)	−0.006 (4)	−0.003 (3)	−0.013 (3)
C11	0.048 (8)	0.054 (10)	0.061 (10)	−0.010 (7)	−0.008 (8)	−0.040 (8)
C12	0.043 (7)	0.042 (8)	0.036 (8)	0.000 (6)	0.006 (5)	−0.017 (7)

### N,N'-(1,4-Phenylene)bis(propan-2-iminium) octa-µ3-chlorido-hexachlorido-octahedro-hexamolybdate acetone trisolvate (3) Geometric parameters (Å, º)

**Table:**

Mo1—Mo2^i^	2.5943 (6)	C2—C3	1.480 (5)
Mo1—Mo2	2.6126 (5)	C3—H3A	0.9800
Mo1—Mo3	2.6038 (6)	C3—H3B	0.9800
Mo1—Mo3^i^	2.6031 (7)	C3—H3C	0.9800
Mo1—Cl2	2.4695 (9)	C4—H4	0.9500
Mo1—Cl3	2.4627 (9)	C4—C5	1.381 (4)
Mo1—Cl4	2.4202 (9)	C4—C6^ii^	1.372 (5)
Mo1—Cl5	2.4727 (9)	C5—C6	1.386 (4)
Mo1—Cl6	2.4729 (9)	C6—H6	0.9500
Mo2—Mo3^i^	2.6069 (6)	O1—C8	1.206 (4)
Mo2—Mo3	2.5989 (7)	C7—H7A	0.9800
Mo2—Cl1	2.4391 (10)	C7—H7B	0.9800
Mo2—Cl2^i^	2.4668 (9)	C7—H7C	0.9800
Mo2—Cl3	2.4727 (9)	C7—C8	1.480 (5)
Mo2—Cl5^i^	2.4738 (9)	C8—C9	1.494 (5)
Mo2—Cl6	2.4616 (9)	C9—H9A	0.9800
Mo3—Cl2^i^	2.4655 (8)	C9—H9B	0.9800
Mo3—Cl3^i^	2.4680 (9)	C9—H9C	0.9800
Mo3—Cl5	2.4767 (8)	O2—C10	1.193 (8)
Mo3—Cl6	2.4714 (9)	C10—C11	1.488 (13)
Mo3—Cl7	2.4110 (10)	C10—C12	1.475 (13)
N1—H1	0.870 (18)	C11—H11A	0.9800
N1—C2	1.284 (4)	C11—H11B	0.9800
N1—C5	1.434 (4)	C11—H11C	0.9800
C1—H1A	0.9800	C12—H12A	0.9800
C1—H1B	0.9800	C12—H12B	0.9800
C1—H1C	0.9800	C12—H12C	0.9800
C1—C2	1.477 (5)		
			
Mo2^i^—Mo1—Mo2	89.98 (2)	Cl3^i^—Mo3—Cl5	89.98 (3)
Mo2^i^—Mo1—Mo3^i^	60.005 (19)	Cl3^i^—Mo3—Cl6	175.12 (3)
Mo2^i^—Mo1—Mo3	60.202 (14)	Cl5—Mo3—Mo1	58.18 (2)
Mo3^i^—Mo1—Mo2	59.977 (16)	Cl5—Mo3—Mo1^i^	118.35 (2)
Mo3—Mo1—Mo2	59.763 (15)	Cl5—Mo3—Mo2^i^	58.17 (2)
Mo3^i^—Mo1—Mo3	89.98 (2)	Cl5—Mo3—Mo2	118.46 (3)
Cl2—Mo1—Mo2	118.05 (2)	Cl6—Mo3—Mo1	58.25 (2)
Cl2—Mo1—Mo2^i^	58.24 (2)	Cl6—Mo3—Mo1^i^	117.84 (3)
Cl2—Mo1—Mo3^i^	58.09 (2)	Cl6—Mo3—Mo2	58.02 (2)
Cl2—Mo1—Mo3	118.42 (3)	Cl6—Mo3—Mo2^i^	117.94 (2)
Cl2—Mo1—Cl5	90.29 (3)	Cl6—Mo3—Cl5	90.12 (3)
Cl2—Mo1—Cl6	175.36 (2)	Cl7—Mo3—Mo1	134.62 (3)
Cl3—Mo1—Mo2	58.22 (2)	Cl7—Mo3—Mo1^i^	135.36 (2)
Cl3—Mo1—Mo2^i^	118.22 (2)	Cl7—Mo3—Mo2^i^	134.89 (3)
Cl3—Mo1—Mo3^i^	58.23 (2)	Cl7—Mo3—Mo2	135.11 (3)
Cl3—Mo1—Mo3	117.98 (2)	Cl7—Mo3—Cl2^i^	92.39 (3)
Cl3—Mo1—Cl2	89.80 (3)	Cl7—Mo3—Cl3^i^	92.59 (3)
Cl3—Mo1—Cl5	175.65 (2)	Cl7—Mo3—Cl5	91.72 (3)
Cl3—Mo1—Cl6	89.39 (3)	Cl7—Mo3—Cl6	92.28 (3)
Cl4—Mo1—Mo2^i^	134.02 (3)	Mo2^i^—Cl2—Mo1	63.41 (2)
Cl4—Mo1—Mo2	136.00 (2)	Mo3^i^—Cl2—Mo1	63.67 (2)
Cl4—Mo1—Mo3^i^	135.46 (3)	Mo3^i^—Cl2—Mo2^i^	63.59 (2)
Cl4—Mo1—Mo3	134.55 (2)	Mo1—Cl3—Mo2	63.92 (2)
Cl4—Mo1—Cl2	91.90 (3)	Mo1—Cl3—Mo3^i^	63.73 (2)
Cl4—Mo1—Cl3	93.21 (3)	Mo3^i^—Cl3—Mo2	63.69 (2)
Cl4—Mo1—Cl5	91.13 (3)	Mo1—Cl5—Mo2^i^	63.26 (2)
Cl4—Mo1—Cl6	92.71 (3)	Mo1—Cl5—Mo3	63.48 (2)
Cl5—Mo1—Mo2^i^	58.39 (2)	Mo2^i^—Cl5—Mo3	63.55 (2)
Cl5—Mo1—Mo2	118.08 (2)	Mo2—Cl6—Mo1	63.94 (2)
Cl5—Mo1—Mo3	58.33 (2)	Mo2—Cl6—Mo3	63.58 (3)
Cl5—Mo1—Mo3^i^	118.37 (2)	Mo3—Cl6—Mo1	63.56 (2)
Cl5—Mo1—Cl6	90.17 (3)	C2—N1—H1	117 (2)
Cl6—Mo1—Mo2^i^	118.37 (2)	C2—N1—C5	129.1 (3)
Cl6—Mo1—Mo2	57.82 (2)	C5—N1—H1	114 (2)
Cl6—Mo1—Mo3	58.19 (2)	H1A—C1—H1B	109.5
Cl6—Mo1—Mo3^i^	117.79 (2)	H1A—C1—H1C	109.5
Mo1^i^—Mo2—Mo1	90.02 (2)	H1B—C1—H1C	109.5
Mo1^i^—Mo2—Mo3	60.165 (17)	C2—C1—H1A	109.5
Mo1^i^—Mo2—Mo3^i^	60.081 (15)	C2—C1—H1B	109.5
Mo3—Mo2—Mo1	59.951 (17)	C2—C1—H1C	109.5
Mo3^i^—Mo2—Mo1	59.830 (17)	N1—C2—C1	118.1 (3)
Mo3—Mo2—Mo3^i^	90.00 (2)	N1—C2—C3	122.8 (3)
Cl1—Mo2—Mo1	135.83 (3)	C1—C2—C3	119.1 (3)
Cl1—Mo2—Mo1^i^	134.15 (2)	C2—C3—H3A	109.5
Cl1—Mo2—Mo3	134.88 (3)	C2—C3—H3B	109.5
Cl1—Mo2—Mo3^i^	135.11 (2)	C2—C3—H3C	109.5
Cl1—Mo2—Cl2^i^	91.56 (3)	H3A—C3—H3B	109.5
Cl1—Mo2—Cl3	93.08 (3)	H3A—C3—H3C	109.5
Cl1—Mo2—Cl5^i^	91.61 (3)	H3B—C3—H3C	109.5
Cl1—Mo2—Cl6	92.55 (3)	C5—C4—H4	120.4
Cl2^i^—Mo2—Mo1^i^	58.35 (2)	C6^ii^—C4—H4	120.4
Cl2^i^—Mo2—Mo1	118.11 (2)	C6^ii^—C4—C5	119.1 (3)
Cl2^i^—Mo2—Mo3^i^	118.40 (2)	C4—C5—N1	121.0 (3)
Cl2^i^—Mo2—Mo3	58.18 (2)	C4—C5—C6	121.2 (3)
Cl2^i^—Mo2—Cl3	175.35 (2)	C6—C5—N1	117.7 (3)
Cl2^i^—Mo2—Cl5^i^	90.33 (3)	C4^ii^—C6—C5	119.6 (3)
Cl3—Mo2—Mo1	57.85 (2)	C4^ii^—C6—H6	120.2
Cl3—Mo2—Mo1^i^	118.13 (2)	C5—C6—H6	120.2
Cl3—Mo2—Mo3^i^	58.07 (2)	H7A—C7—H7B	109.5
Cl3—Mo2—Mo3	117.79 (2)	H7A—C7—H7C	109.5
Cl3—Mo2—Cl5^i^	89.93 (3)	H7B—C7—H7C	109.5
Cl5^i^—Mo2—Mo1	118.09 (2)	C8—C7—H7A	109.5
Cl5^i^—Mo2—Mo1^i^	58.35 (2)	C8—C7—H7B	109.5
Cl5^i^—Mo2—Mo3^i^	58.28 (2)	C8—C7—H7C	109.5
Cl5^i^—Mo2—Mo3	118.49 (2)	O1—C8—C7	121.9 (4)
Cl6—Mo2—Mo1	58.24 (2)	O1—C8—C9	120.5 (3)
Cl6—Mo2—Mo1^i^	118.54 (3)	C7—C8—C9	117.7 (3)
Cl6—Mo2—Mo3^i^	118.06 (2)	C8—C9—H9A	109.5
Cl6—Mo2—Mo3	58.39 (2)	C8—C9—H9B	109.5
Cl6—Mo2—Cl2^i^	89.98 (3)	C8—C9—H9C	109.5
Cl6—Mo2—Cl3	89.42 (3)	H9A—C9—H9B	109.5
Cl6—Mo2—Cl5^i^	175.82 (2)	H9A—C9—H9C	109.5
Mo1^i^—Mo3—Mo1	90.025 (19)	H9B—C9—H9C	109.5
Mo1—Mo3—Mo2^i^	59.719 (18)	O2—C10—C11	122.1 (9)
Mo1^i^—Mo3—Mo2^i^	60.193 (13)	O2—C10—C12	120.8 (9)
Mo2—Mo3—Mo1	60.287 (13)	C12—C10—C11	116.9 (14)
Mo2—Mo3—Mo1^i^	59.830 (12)	C10—C11—H11A	109.5
Mo2—Mo3—Mo2^i^	90.00 (2)	C10—C11—H11B	109.5
Cl2^i^—Mo3—Mo1	118.50 (2)	C10—C11—H11C	109.5
Cl2^i^—Mo3—Mo1^i^	58.24 (2)	H11A—C11—H11B	109.5
Cl2^i^—Mo3—Mo2	58.23 (2)	H11A—C11—H11C	109.5
Cl2^i^—Mo3—Mo2^i^	118.42 (2)	H11B—C11—H11C	109.5
Cl2^i^—Mo3—Cl3^i^	89.77 (3)	C10—C12—H12A	109.5
Cl2^i^—Mo3—Cl5	175.88 (3)	C10—C12—H12B	109.5
Cl2^i^—Mo3—Cl6	89.79 (3)	C10—C12—H12C	109.5
Cl3^i^—Mo3—Mo1^i^	58.03 (2)	H12A—C12—H12B	109.5
Cl3^i^—Mo3—Mo1	117.94 (2)	H12A—C12—H12C	109.5
Cl3^i^—Mo3—Mo2^i^	58.24 (2)	H12B—C12—H12C	109.5
Cl3^i^—Mo3—Mo2	117.85 (2)		
			
N1—C5—C6—C4^ii^	176.9 (3)	C5—N1—C2—C1	174.4 (3)
C2—N1—C5—C4	−46.6 (5)	C5—N1—C2—C3	−4.4 (5)
C2—N1—C5—C6	136.5 (3)	C6^ii^—C4—C5—N1	−176.7 (3)
C4—C5—C6—C4^ii^	−0.1 (5)	C6^ii^—C4—C5—C6	0.1 (5)

Symmetry codes: (i) −*x*+1, −*y*+1, −*z*+1; (ii) −*x*, −*y*, −*z*+1.

### N,N'-(1,4-Phenylene)bis(propan-2-iminium) octa-µ3-chlorido-hexachlorido-octahedro-hexamolybdate acetone trisolvate (3) Hydrogen-bond geometry (Å, º)

**Table:**

*D*—H···*A*	*D*—H	H···*A*	*D*···*A*	*D*—H···*A*
N1—H1···O1^iii^	0.87 (2)	1.93 (2)	2.791 (4)	172 (3)

Symmetry code: (iii) *x*, *y*−1, *z*.

### 1,1'-Dimethyl-4,4'-bipyridinium octa-µ3-chlorido-hexachlorido-octahedro-hexamolybdate N,N-dimethylformamide tetrasolvate (4) Crystal data

**Table:**

(C_12_H_14_N_2_)[Mo_6_Cl_8_Cl_6_]·4C_3_H_7_NO	*Z* = 1
*M**_r_* = 1550.57	*F*(000) = 750
Triclinic, *P*1	*D*_x_ = 2.161 Mg m^−^^3^
*a* = 9.8252 (11) Å	Mo *K*α radiation, λ = 0.71073 Å
*b* = 10.0933 (11) Å	Cell parameters from 6604 reflections
*c* = 12.6319 (15) Å	θ = 2.6–25.0°
α = 107.395 (3)°	µ = 2.35 mm^−^^1^
β = 91.881 (3)°	*T* = 200 K
γ = 93.309 (3)°	Block, orange
*V* = 1191.8 (2) Å^3^	0.32 × 0.30 × 0.28 mm

### 1,1'-Dimethyl-4,4'-bipyridinium octa-µ3-chlorido-hexachlorido-octahedro-hexamolybdate N,N-dimethylformamide tetrasolvate (4) Data collection

**Table:**

Bruker SMART X2S benchtop diffractometer	4187 independent reflections
Radiation source: sealed microfocus source, XOS X-beam microfocus source	3743 reflections with *I* > 2σ(*I*)
Graphite monochromator	*R*_int_ = 0.024
Detector resolution: 8.3330 pixels mm^-1^	θ_max_ = 25.1°, θ_min_ = 2.3°
φ and ω scans	*h* = −11→11
Absorption correction: multi-scan (SADABS; Bruker, 2012)	*k* = −11→11
*T*_min_ = 0.815, *T*_max_ = 1.000	*l* = −15→15
11498 measured reflections	

### 1,1'-Dimethyl-4,4'-bipyridinium octa-µ3-chlorido-hexachlorido-octahedro-hexamolybdate N,N-dimethylformamide tetrasolvate (4) Refinement

**Table:**

Refinement on *F*^2^	Hydrogen site location: inferred from neighbouring sites
Least-squares matrix: full	H-atom parameters constrained
*R*[*F*^2^ > 2σ(*F*^2^)] = 0.020	*w* = 1/[σ^2^(*F*_o_^2^) + (0.0184*P*)^2^ + 0.8263*P*] where *P* = (*F*_o_^2^ + 2*F*_c_^2^)/3
*wR*(*F*^2^) = 0.049	(Δ/σ)_max_ = 0.002
*S* = 1.02	Δρ_max_ = 0.42 e Å^−^^3^
4187 reflections	Δρ_min_ = −0.44 e Å^−^^3^
250 parameters	Extinction correction: SHELXL2018 (Sheldrick, 2015), Fc^*^=kFc[1+0.001xFc^2^λ^3^/sin(2θ)]^-1/4^
0 restraints	Extinction coefficient: 0.0075 (3)
Primary atom site location: heavy-atom method	

### 1,1'-Dimethyl-4,4'-bipyridinium octa-µ3-chlorido-hexachlorido-octahedro-hexamolybdate N,N-dimethylformamide tetrasolvate (4) Special details

**Table:**

Geometry. All esds (except the esd in the dihedral angle between two l.s. planes) are estimated using the full covariance matrix. The cell esds are taken into account individually in the estimation of esds in distances, angles and torsion angles; correlations between esds in cell parameters are only used when they are defined by crystal symmetry. An approximate (isotropic) treatment of cell esds is used for estimating esds involving l.s. planes.

### 1,1'-Dimethyl-4,4'-bipyridinium octa-µ3-chlorido-hexachlorido-octahedro-hexamolybdate N,N-dimethylformamide tetrasolvate (4) Fractional atomic coordinates and isotropic or equivalent isotropic displacement parameters (Å^2^)

**Table:**

	*x*	*y*	*z*	*U*_iso_*/*U*_eq_	
Mo1	0.34350 (2)	0.52105 (2)	0.42443 (2)	0.01878 (7)	
Mo2	0.43880 (2)	0.61443 (2)	0.62913 (2)	0.01930 (7)	
Mo3	0.41622 (2)	0.34763 (2)	0.52876 (2)	0.01916 (7)	
Cl4	0.21336 (6)	0.48336 (6)	0.57792 (5)	0.02520 (14)	
Cl2	0.51113 (6)	0.44527 (7)	0.72253 (5)	0.02551 (14)	
Cl7	0.30745 (7)	0.14843 (7)	0.56719 (6)	0.03486 (17)	
Cl6	0.35663 (7)	0.76331 (7)	0.79813 (6)	0.03874 (18)	
Cl3	0.32970 (6)	0.26780 (6)	0.33243 (5)	0.02516 (14)	
Cl1	0.37330 (6)	0.77313 (6)	0.52328 (5)	0.02510 (14)	
Cl5	0.13401 (6)	0.54800 (7)	0.32618 (5)	0.03256 (16)	
N1	0.0168 (2)	0.9093 (3)	0.7467 (2)	0.0392 (6)	
C3	0.0031 (2)	0.9806 (3)	0.5519 (2)	0.0299 (6)	
C6	0.0223 (4)	0.8700 (4)	0.8508 (3)	0.0560 (9)	
H6A	0.092144	0.930439	0.903109	0.084*	
H6B	0.045141	0.772867	0.834244	0.084*	
H6C	−0.066769	0.880619	0.883944	0.084*	
O1	0.0987 (4)	0.2529 (3)	0.9699 (2)	0.0947 (11)	
N2	0.1930 (3)	0.4599 (3)	0.9693 (2)	0.0462 (7)	
C7	0.1610 (5)	0.3616 (4)	1.0150 (3)	0.0661 (11)	
H7	0.190127	0.378592	1.090665	0.079*	
C9	0.1539 (4)	0.4414 (4)	0.8553 (3)	0.0642 (10)	
H9A	0.191569	0.357250	0.808268	0.096*	
H9B	0.189323	0.522299	0.834281	0.096*	
H9C	0.054111	0.432088	0.845257	0.096*	
C8	0.2613 (6)	0.5918 (5)	1.0316 (4)	0.0954 (17)	
H8A	0.274154	0.595293	1.109641	0.143*	
H8B	0.205703	0.667344	1.026178	0.143*	
H8C	0.350353	0.602489	1.001246	0.143*	
O2	0.2430 (3)	0.9764 (3)	0.1097 (2)	0.0629 (7)	
N3	0.4369 (3)	0.9015 (3)	0.1660 (2)	0.0425 (6)	
C10	0.3573 (4)	0.9392 (3)	0.0942 (3)	0.0479 (8)	
H10	0.393572	0.936671	0.024855	0.058*	
C12	0.3883 (4)	0.9057 (4)	0.2736 (3)	0.0567 (9)	
H12A	0.311583	0.965299	0.289780	0.085*	
H12B	0.462220	0.943321	0.330545	0.085*	
H12C	0.358102	0.811373	0.273342	0.085*	
C11	0.5716 (4)	0.8576 (4)	0.1409 (4)	0.0698 (11)	
H11A	0.576876	0.762359	0.144373	0.105*	
H11B	0.638360	0.920002	0.195053	0.105*	
H11C	0.591481	0.860583	0.066069	0.105*	
C2	0.0219 (3)	0.8454 (3)	0.5522 (2)	0.0407 (7)	
H2	0.030656	0.774946	0.483942	0.049*	
C1	0.0281 (3)	0.8128 (4)	0.6492 (3)	0.0450 (8)	
H1	0.040636	0.719562	0.647548	0.054*	
C4	−0.0086 (4)	1.0768 (3)	0.6542 (3)	0.0499 (9)	
H4	−0.021238	1.170823	0.658585	0.060*	
C5	−0.0023 (4)	1.0392 (3)	0.7493 (3)	0.0539 (9)	
H5	−0.011812	1.107404	0.818648	0.065*	

### 1,1'-Dimethyl-4,4'-bipyridinium octa-µ3-chlorido-hexachlorido-octahedro-hexamolybdate N,N-dimethylformamide tetrasolvate (4) Atomic displacement parameters (Å^2^)

**Table:**

	*U*^11^	*U*^22^	*U*^33^	*U*^12^	*U*^13^	*U*^23^
Mo1	0.01652 (11)	0.02000 (13)	0.02050 (12)	0.00181 (8)	0.00057 (8)	0.00707 (9)
Mo2	0.01846 (11)	0.01990 (13)	0.01947 (12)	0.00228 (9)	0.00309 (8)	0.00547 (9)
Mo3	0.01755 (11)	0.01909 (13)	0.02210 (12)	0.00020 (8)	0.00183 (8)	0.00827 (9)
Cl4	0.0185 (3)	0.0295 (3)	0.0290 (3)	0.0021 (2)	0.0059 (2)	0.0103 (3)
Cl2	0.0272 (3)	0.0301 (4)	0.0223 (3)	0.0025 (3)	0.0018 (2)	0.0124 (3)
Cl7	0.0300 (3)	0.0327 (4)	0.0474 (4)	−0.0075 (3)	−0.0014 (3)	0.0226 (3)
Cl6	0.0430 (4)	0.0374 (4)	0.0305 (4)	0.0072 (3)	0.0131 (3)	0.0001 (3)
Cl3	0.0239 (3)	0.0227 (3)	0.0259 (3)	−0.0016 (2)	−0.0023 (2)	0.0038 (3)
Cl1	0.0240 (3)	0.0207 (3)	0.0318 (3)	0.0047 (2)	0.0021 (2)	0.0090 (3)
Cl5	0.0254 (3)	0.0383 (4)	0.0335 (4)	0.0070 (3)	−0.0058 (3)	0.0100 (3)
N1	0.0294 (12)	0.0537 (17)	0.0346 (13)	0.0046 (11)	0.0096 (10)	0.0125 (12)
C3	0.0193 (12)	0.0339 (16)	0.0316 (14)	−0.0056 (11)	0.0078 (11)	0.0031 (12)
C6	0.054 (2)	0.082 (3)	0.0421 (19)	0.0242 (19)	0.0164 (16)	0.0289 (19)
O1	0.166 (3)	0.0601 (18)	0.0529 (17)	−0.046 (2)	0.0022 (19)	0.0192 (15)
N2	0.0566 (17)	0.0467 (16)	0.0351 (14)	−0.0092 (13)	−0.0047 (12)	0.0153 (13)
C7	0.105 (3)	0.061 (3)	0.0329 (18)	−0.010 (2)	0.0046 (19)	0.0184 (19)
C9	0.078 (3)	0.070 (3)	0.047 (2)	−0.013 (2)	−0.0116 (19)	0.028 (2)
C8	0.132 (4)	0.080 (3)	0.067 (3)	−0.046 (3)	−0.033 (3)	0.026 (3)
O2	0.0563 (16)	0.0770 (18)	0.0618 (16)	0.0167 (14)	−0.0073 (13)	0.0295 (14)
N3	0.0483 (15)	0.0320 (14)	0.0497 (16)	0.0033 (12)	−0.0034 (13)	0.0165 (12)
C10	0.060 (2)	0.0404 (19)	0.0432 (18)	−0.0019 (16)	−0.0034 (16)	0.0146 (16)
C12	0.078 (3)	0.048 (2)	0.047 (2)	0.0120 (18)	−0.0094 (18)	0.0183 (17)
C11	0.056 (2)	0.064 (3)	0.100 (3)	0.0104 (19)	0.009 (2)	0.038 (2)
C2	0.0383 (16)	0.0433 (19)	0.0360 (16)	0.0171 (14)	0.0025 (13)	0.0024 (14)
C1	0.0423 (17)	0.049 (2)	0.0455 (19)	0.0226 (15)	0.0062 (14)	0.0125 (16)
C4	0.079 (2)	0.0295 (17)	0.0366 (17)	−0.0111 (16)	0.0227 (16)	0.0043 (14)
C5	0.080 (3)	0.039 (2)	0.0360 (18)	−0.0106 (17)	0.0218 (17)	0.0016 (15)

### 1,1'-Dimethyl-4,4'-bipyridinium octa-µ3-chlorido-hexachlorido-octahedro-hexamolybdate N,N-dimethylformamide tetrasolvate (4) Geometric parameters (Å, º)

**Table:**

Mo1—Mo2^i^	2.6043 (4)	C6—H6C	0.9800
Mo1—Mo2	2.5948 (4)	O1—C7	1.195 (4)
Mo1—Mo3	2.6026 (3)	N2—C7	1.318 (4)
Mo1—Mo3^i^	2.5996 (4)	N2—C9	1.432 (4)
Mo1—Cl4	2.4671 (6)	N2—C8	1.442 (5)
Mo1—Cl2^i^	2.4692 (6)	C7—H7	0.9500
Mo1—Cl3	2.4650 (7)	C9—H9A	0.9800
Mo1—Cl1	2.4716 (7)	C9—H9B	0.9800
Mo1—Cl5	2.4392 (7)	C9—H9C	0.9800
Mo2—Mo3^i^	2.5949 (3)	C8—H8A	0.9800
Mo2—Mo3	2.6037 (4)	C8—H8B	0.9800
Mo2—Cl4	2.4760 (6)	C8—H8C	0.9800
Mo2—Cl2	2.4701 (6)	O2—C10	1.208 (4)
Mo2—Cl6	2.4088 (7)	N3—C10	1.331 (4)
Mo2—Cl3^i^	2.4683 (6)	N3—C12	1.445 (4)
Mo2—Cl1	2.4720 (6)	N3—C11	1.435 (4)
Mo3—Cl4	2.4745 (6)	C10—H10	0.9500
Mo3—Cl2	2.4766 (7)	C12—H12A	0.9800
Mo3—Cl7	2.4061 (7)	C12—H12B	0.9800
Mo3—Cl3	2.4719 (7)	C12—H12C	0.9800
Mo3—Cl1^i^	2.4689 (6)	C11—H11A	0.9800
N1—C6	1.484 (4)	C11—H11B	0.9800
N1—C1	1.334 (4)	C11—H11C	0.9800
N1—C5	1.327 (4)	C2—H2	0.9500
C3—C3^ii^	1.477 (5)	C2—C1	1.361 (4)
C3—C2	1.388 (4)	C1—H1	0.9500
C3—C4	1.377 (4)	C4—H4	0.9500
C6—H6A	0.9800	C4—C5	1.363 (4)
C6—H6B	0.9800	C5—H5	0.9500
			
Mo2—Mo1—Mo2^i^	89.921 (11)	Cl7—Mo3—Mo2^i^	135.04 (2)
Mo2—Mo1—Mo3^i^	59.942 (8)	Cl7—Mo3—Cl4	92.33 (2)
Mo2—Mo1—Mo3	60.128 (10)	Cl7—Mo3—Cl2	92.13 (2)
Mo3—Mo1—Mo2^i^	59.784 (9)	Cl7—Mo3—Cl3	92.61 (2)
Mo3^i^—Mo1—Mo2^i^	60.045 (11)	Cl7—Mo3—Cl1^i^	91.81 (2)
Mo3^i^—Mo1—Mo3	89.992 (11)	Cl3—Mo3—Mo1	58.057 (16)
Cl4—Mo1—Mo2^i^	118.129 (17)	Cl3—Mo3—Mo1^i^	118.159 (16)
Cl4—Mo1—Mo2	58.503 (17)	Cl3—Mo3—Mo2	117.829 (17)
Cl4—Mo1—Mo3^i^	118.428 (17)	Cl3—Mo3—Mo2^i^	58.247 (16)
Cl4—Mo1—Mo3	58.357 (16)	Cl3—Mo3—Cl4	89.57 (2)
Cl4—Mo1—Cl2^i^	175.80 (2)	Cl3—Mo3—Cl2	175.25 (2)
Cl4—Mo1—Cl1	90.24 (2)	Cl1^i^—Mo3—Mo1	118.500 (16)
Cl2^i^—Mo1—Mo2	118.347 (18)	Cl1^i^—Mo3—Mo1^i^	58.302 (17)
Cl2^i^—Mo1—Mo2^i^	58.200 (16)	Cl1^i^—Mo3—Mo2^i^	58.376 (16)
Cl2^i^—Mo1—Mo3^i^	58.426 (16)	Cl1^i^—Mo3—Mo2	118.352 (17)
Cl2^i^—Mo1—Mo3	117.975 (17)	Cl1^i^—Mo3—Cl4	175.86 (2)
Cl2^i^—Mo1—Cl1	90.06 (2)	Cl1^i^—Mo3—Cl2	89.95 (2)
Cl3—Mo1—Mo2^i^	58.199 (15)	Cl1^i^—Mo3—Cl3	90.20 (2)
Cl3—Mo1—Mo2	118.426 (17)	Mo1—Cl4—Mo2	63.328 (16)
Cl3—Mo1—Mo3	58.314 (17)	Mo1—Cl4—Mo3	63.562 (15)
Cl3—Mo1—Mo3^i^	118.235 (16)	Mo3—Cl4—Mo2	63.465 (17)
Cl3—Mo1—Cl4	89.90 (2)	Mo1^i^—Cl2—Mo2	63.638 (16)
Cl3—Mo1—Cl2^i^	89.50 (2)	Mo1^i^—Cl2—Mo3	63.422 (17)
Cl3—Mo1—Cl1	175.83 (2)	Mo2—Cl2—Mo3	63.519 (17)
Cl1—Mo1—Mo2^i^	118.234 (17)	Mo1—Cl3—Mo2^i^	63.726 (16)
Cl1—Mo1—Mo2	58.347 (15)	Mo1—Cl3—Mo3	63.629 (16)
Cl1—Mo1—Mo3^i^	58.203 (15)	Mo2^i^—Cl3—Mo3	63.372 (16)
Cl1—Mo1—Mo3	118.456 (18)	Mo1—Cl1—Mo2	63.322 (17)
Cl5—Mo1—Mo2	134.428 (19)	Mo3^i^—Cl1—Mo1	63.496 (16)
Cl5—Mo1—Mo2^i^	135.644 (19)	Mo3^i^—Cl1—Mo2	63.363 (16)
Cl5—Mo1—Mo3	134.380 (19)	C1—N1—C6	119.9 (3)
Cl5—Mo1—Mo3^i^	135.62 (2)	C5—N1—C6	120.5 (3)
Cl5—Mo1—Cl4	91.17 (2)	C5—N1—C1	119.6 (3)
Cl5—Mo1—Cl2^i^	93.01 (2)	C2—C3—C3^ii^	122.0 (3)
Cl5—Mo1—Cl3	92.03 (2)	C4—C3—C3^ii^	121.9 (3)
Cl5—Mo1—Cl1	92.13 (2)	C4—C3—C2	116.1 (3)
Mo1—Mo2—Mo1^i^	90.078 (10)	N1—C6—H6A	109.5
Mo1—Mo2—Mo3^i^	60.121 (11)	N1—C6—H6B	109.5
Mo1—Mo2—Mo3	60.084 (8)	N1—C6—H6C	109.5
Mo3—Mo2—Mo1^i^	59.890 (9)	H6A—C6—H6B	109.5
Mo3^i^—Mo2—Mo1^i^	60.076 (9)	H6A—C6—H6C	109.5
Mo3^i^—Mo2—Mo3	90.070 (10)	H6B—C6—H6C	109.5
Cl4—Mo2—Mo1^i^	118.113 (18)	C7—N2—C9	120.4 (3)
Cl4—Mo2—Mo1	58.169 (15)	C7—N2—C8	122.3 (3)
Cl4—Mo2—Mo3	58.238 (16)	C9—N2—C8	117.2 (3)
Cl4—Mo2—Mo3^i^	118.272 (17)	O1—C7—N2	125.8 (3)
Cl2—Mo2—Mo1	118.429 (18)	O1—C7—H7	117.1
Cl2—Mo2—Mo1^i^	58.164 (15)	N2—C7—H7	117.1
Cl2—Mo2—Mo3	58.360 (17)	N2—C9—H9A	109.5
Cl2—Mo2—Mo3^i^	118.230 (17)	N2—C9—H9B	109.5
Cl2—Mo2—Cl4	90.05 (2)	N2—C9—H9C	109.5
Cl2—Mo2—Cl1	175.86 (2)	H9A—C9—H9B	109.5
Cl6—Mo2—Mo1	134.52 (2)	H9A—C9—H9C	109.5
Cl6—Mo2—Mo1^i^	135.40 (2)	H9B—C9—H9C	109.5
Cl6—Mo2—Mo3	134.68 (2)	N2—C8—H8A	109.5
Cl6—Mo2—Mo3^i^	135.25 (2)	N2—C8—H8B	109.5
Cl6—Mo2—Cl4	91.70 (2)	N2—C8—H8C	109.5
Cl6—Mo2—Cl2	92.13 (2)	H8A—C8—H8B	109.5
Cl6—Mo2—Cl3^i^	92.69 (2)	H8A—C8—H8C	109.5
Cl6—Mo2—Cl1	92.01 (3)	H8B—C8—H8C	109.5
Cl3^i^—Mo2—Mo1^i^	58.075 (17)	C10—N3—C12	119.7 (3)
Cl3^i^—Mo2—Mo1	118.479 (16)	C10—N3—C11	122.2 (3)
Cl3^i^—Mo2—Mo3^i^	58.382 (16)	C11—N3—C12	118.1 (3)
Cl3^i^—Mo2—Mo3	117.956 (16)	O2—C10—N3	125.6 (3)
Cl3^i^—Mo2—Cl4	175.60 (2)	O2—C10—H10	117.2
Cl3^i^—Mo2—Cl2	89.40 (2)	N3—C10—H10	117.2
Cl3^i^—Mo2—Cl1	90.21 (2)	N3—C12—H12A	109.5
Cl1—Mo2—Mo1^i^	118.322 (16)	N3—C12—H12B	109.5
Cl1—Mo2—Mo1	58.332 (17)	N3—C12—H12C	109.5
Cl1—Mo2—Mo3^i^	58.262 (16)	H12A—C12—H12B	109.5
Cl1—Mo2—Mo3	118.397 (19)	H12A—C12—H12C	109.5
Cl1—Mo2—Cl4	90.03 (2)	H12B—C12—H12C	109.5
Mo1^i^—Mo3—Mo1	90.008 (11)	N3—C11—H11A	109.5
Mo1^i^—Mo3—Mo2	60.064 (8)	N3—C11—H11B	109.5
Mo1—Mo3—Mo2	59.788 (11)	N3—C11—H11C	109.5
Mo2^i^—Mo3—Mo1	60.140 (9)	H11A—C11—H11B	109.5
Mo2^i^—Mo3—Mo1^i^	59.936 (10)	H11A—C11—H11C	109.5
Mo2^i^—Mo3—Mo2	89.930 (10)	H11B—C11—H11C	109.5
Cl4—Mo3—Mo1^i^	118.345 (18)	C3—C2—H2	119.6
Cl4—Mo3—Mo1	58.082 (15)	C1—C2—C3	120.8 (3)
Cl4—Mo3—Mo2	58.296 (16)	C1—C2—H2	119.6
Cl4—Mo3—Mo2^i^	118.209 (17)	N1—C1—C2	121.2 (3)
Cl4—Mo3—Cl2	89.94 (2)	N1—C1—H1	119.4
Cl2—Mo3—Mo1	117.894 (18)	C2—C1—H1	119.4
Cl2—Mo3—Mo1^i^	58.152 (15)	C3—C4—H4	119.4
Cl2—Mo3—Mo2^i^	118.066 (17)	C5—C4—C3	121.1 (3)
Cl2—Mo3—Mo2	58.121 (16)	C5—C4—H4	119.4
Cl7—Mo3—Mo1	135.303 (19)	N1—C5—C4	121.2 (3)
Cl7—Mo3—Mo1^i^	134.689 (19)	N1—C5—H5	119.4
Cl7—Mo3—Mo2	135.02 (2)	C4—C5—H5	119.4
			
C3^ii^—C3—C2—C1	179.5 (3)	C8—N2—C7—O1	176.1 (5)
C3^ii^—C3—C4—C5	−179.8 (3)	C12—N3—C10—O2	−1.1 (5)
C3—C2—C1—N1	−0.3 (5)	C11—N3—C10—O2	179.6 (3)
C3—C4—C5—N1	0.8 (5)	C2—C3—C4—C5	−0.3 (5)
C6—N1—C1—C2	179.3 (3)	C1—N1—C5—C4	−1.0 (5)
C6—N1—C5—C4	−179.5 (3)	C4—C3—C2—C1	0.0 (4)
C9—N2—C7—O1	−0.6 (7)	C5—N1—C1—C2	0.8 (5)

Symmetry codes: (i) −*x*+1, −*y*+1, −*z*+1; (ii) −*x*, −*y*+2, −*z*+1.

### 1,1'-Dimethyl-4,4'-bipyridinium octa-µ3-chlorido-hexachlorido-octahedro-hexamolybdate N,N-dimethylformamide tetrasolvate (4) Hydrogen-bond geometry (Å, º)

**Table:**

*D*—H···*A*	*D*—H	H···*A*	*D*···*A*	*D*—H···*A*
C5—H5···O1^iii^	0.95	2.23	3.063 (4)	145
C6—H6*C*···O2^ii^	0.98	2.31	3.088 (4)	136

Symmetry codes: (ii) −*x*, −*y*+2, −*z*+1; (iii) *x*, *y*+1, *z*.
